# Membrane Association Landscape of Myelin Basic Protein Portrays Formation of the Myelin Major Dense Line

**DOI:** 10.1038/s41598-017-05364-3

**Published:** 2017-07-10

**Authors:** Arne Raasakka, Salla Ruskamo, Julia Kowal, Robert Barker, Anne Baumann, Anne Martel, Jussi Tuusa, Matti Myllykoski, Jochen Bürck, Anne S. Ulrich, Henning Stahlberg, Petri Kursula

**Affiliations:** 1 Department of Biomedicine, University of Bergen, Bergen, Norway; 2 Faculty of Biochemistry and Molecular Medicine & Biocenter Oulu, University of Oulu, Oulu, Finland; 3 Center for Cellular Imaging and NanoAnalytics (C-CINA), Biozentrum, University of Basel, Basel, Switzerland; 4 School of Physical Sciences, University of Kent, Canterbury, Kent United Kingdom; 5 Institut Laue-Langevin (ILL), Grenoble, France; 6 Division of Psychiatry, Haukeland University Hospital, Bergen, Norway; 7 Institute of Biological Interfaces (IBG-2), Karlsruhe Institute of Technology, Karlsruhe, Germany; 8 Institute of Organic Chemistry, Karlsruhe Institute of Technology, Karlsruhe, Germany

## Abstract

Compact myelin comprises most of the dry weight of myelin, and its insulative nature is the basis for saltatory conduction of nerve impulses. The major dense line (MDL) is a 3-nm compartment between two cytoplasmic leaflets of stacked myelin membranes, mostly occupied by a myelin basic protein (MBP) phase. MBP is an abundant myelin protein involved in demyelinating diseases, such as multiple sclerosis. The association of MBP with lipid membranes has been studied for decades, but the MBP-driven formation of the MDL remains elusive at the biomolecular level. We employed complementary biophysical methods, including atomic force microscopy, cryo-electron microscopy, and neutron scattering, to investigate the formation of membrane stacks all the way from MBP binding onto a single membrane leaflet to the organisation of a stable MDL. Our results support the formation of an amorphous protein phase of MBP between two membrane bilayers and provide a molecular model for MDL formation during myelination, which is of importance when understanding myelin assembly and demyelinating conditions.

## Introduction

Compact myelin (CM) is the most important and abundant structure of the vertebrate myelin sheath in both the central and peripheral nervous systems (CNS and PNS, respectively). The foundation of myelin-accelerated saltatory conduction lies in the insulative nature of CM, which can be disturbed by damage caused by de- or dysmyelination, as well as in the myelin-guided distribution of ion channels on the axonal plasma membrane. Myelin damage often results in chronic neurological conditions, such as multiple sclerosis (MS), Charcot-Marie-Tooth disease, or Dejerine-Sottas syndrome, all of which display a broad spectrum of symptoms, have at least a partial genetic background, and remain difficult to treat, even at an early onset^1, 2^.

Myelin basic protein (MBP) is one of the crucial factors in CM membrane stacking in the CNS, its 18.5-kDa isoform being most abundant^3^. The presence of many MBP isoforms is further complicated through post-translational modifications, including deimination, which produces a pool of citrullinated variants with decreased net charge^4^. The high positive net charge of MBP is related to the intrinsically disordered conformation of MBP in solution; on the other hand, it allows MBP to interact with the phospholipid-rich cytoplasmic face of myelin membranes. This close interaction results in charge neutralisation, folding, and partial membrane insertion^5^. MBP promotes myelin membrane stacking and the formation of the major dense line (MDL), which is disturbed in demyelinating conditions, including MS and demyelinating neuropathies^6, 7^.

MBP is known for its autoantigenic properties in MS, and the major immunodominant epitope of MBP bound to a T-cell receptor complex has been structurally characterised^5, 8^. The autoantigenic character may arise from the susceptibility of MBP to proteolysis in a lipid composition-dependent manner^5, 9^. Additionally, deimination alters the structure and function of MBP, promoting lowered CM stability and increased protease susceptibility^10, 11^. Hence, the membrane association mode, structure, and stability of MBP must be considered when investigating the molecular mechanisms of MBP-related diseases.

MBP has been suggested to form an ordered, self-assembled protein meshwork of either anti-parallel or stacked MBP molecules with confined degrees of freedom between myelin cytoplasmic leaflets^9, 12, 13^. This protein meshwork stabilises CM, while other factors, such as the myelin protein P2, may be involved in the process^14, 15^. The surface adsorption behaviour of MBP has been intensively studied, and it was proposed that MBP associates with the membrane surface prior to folding to its adhesive conformation^16^. This model, based on hard model surfaces, is in corroboration with earlier studies on lipid bilayers^17^, and a mostly disordered intermediate MBP folding state has been proposed based on titration experiments and modelling under conditions with decreasing dielectric constant^18^.

To elucidate the membrane association mechanisms of MBP, we performed a comprehensive characterisation of recombinant tag-free MBP (rMBP) binding to model membranes. Our combined approach using electron microscopy (EM), atomic force microscopy (AFM), and neutron reflectometry (NR) provides evidence for the formation of a dense protein phase on a single membrane leaflet, suggesting the existence of a protein meshwork^13^ that forms above a critical MBP concentration. Our results illustrate a step-wise formation of the MDL at the biomolecular scale.

## Results

Although much is known about the molecular properties of MBP and its interaction with membranes, a comprehensive picture of different steps in the process of myelin membrane compaction has been lacking. We set out to investigate the fine details of MBP-membrane interactions using a panel of biophysical methods to follow membrane binding, protein embedding, and bilayer stacking.

### Characterisation of untagged rMBP

A notable amount of past MBP research has been performed using C-terminally His_6_-tagged recombinant MBP (MBP-His)^12, 14, 15, 19, 20^ or MBP purified from nerve tissue^4, 9, 10, 16, 17, 21^. To overcome problems arising from construct design or contaminants and heterogeneity in tissue extracts, we used untagged recombinant murine MBP (rMBP) corresponding to the major 18.5-kDa isoform, similarly to other recent studies^18, 22–24^. rMBP appeared as a single band in denaturing gel electrophoresis (SDS-PAGE) and monomeric and monodisperse in size-exclusion chromatography (SEC) and dynamic light scattering (DLS), with a hydrodynamic radius (*R*_h_) of 3.5 nm (Supplementary Fig. S1). The identity of rMBP was confirmed using tryptic peptide mass analysis, and the molecular weight of pure rMBP was as expected (18544 Da).

**Figure 1:**
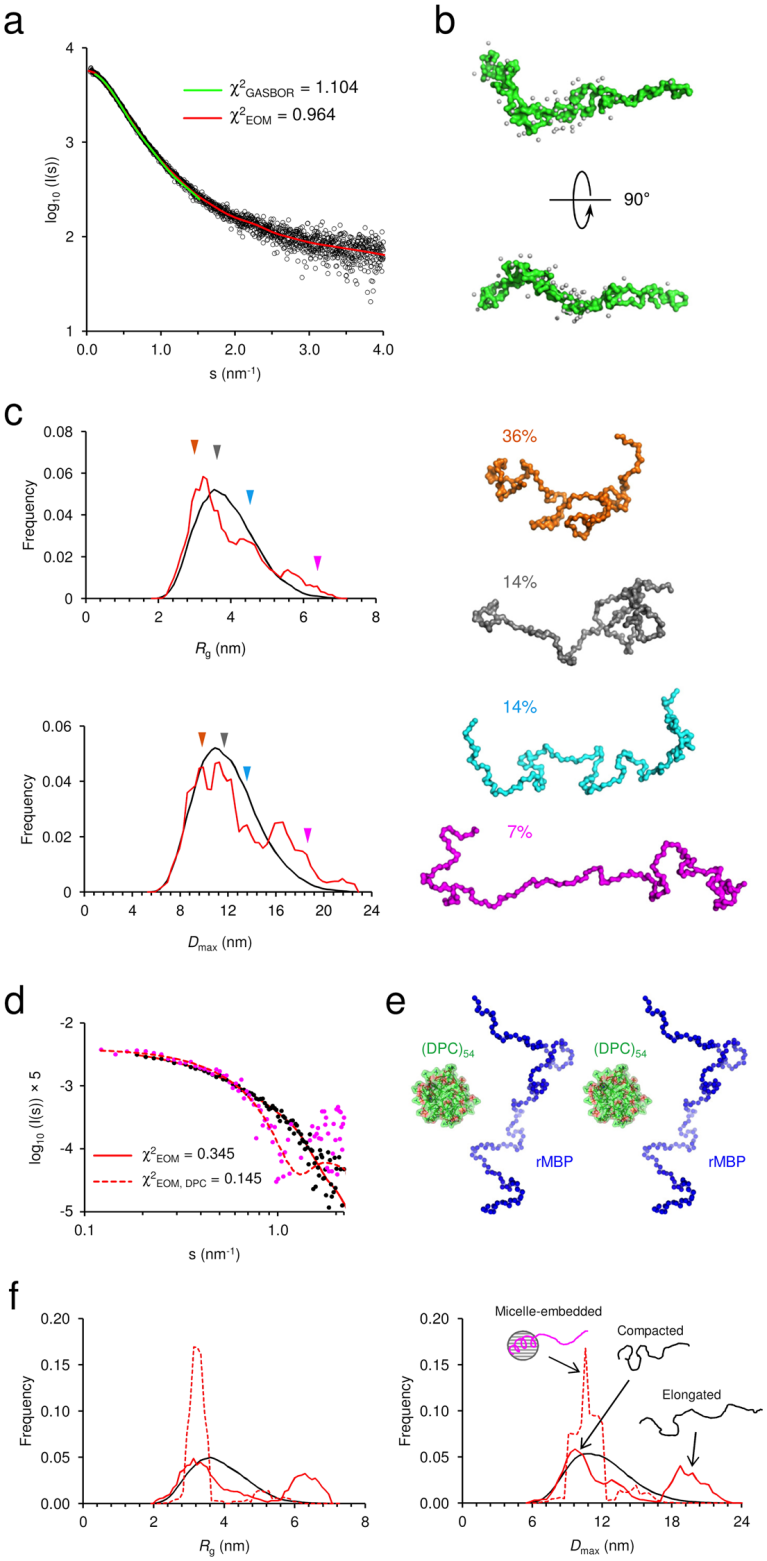
rMBP conformation in solution. (**a**) rMBP is significantly elongated based on SAXS data. GASBOR and EOM fits have been plotted over the raw data with their respective χ^2^ values indicated. (**b**) The GASBOR *ab initio* model is elongated, with a maximum dimension around 12 nm. (**c**) EOM analysis of rMBP reveals wide *R*_g_ and *D*_max_ populations in an ensemble that represents the measured SAXS data. Selected models from EOM analysis (right) with their mass fractions within the total population are indicated. The coloured arrows denote the *R*_g_ and *D*_max_ of each model within the distributions. (**d**) SANS curves of rMBP in the absence and presence of DPC micelles (black and magenta, respectively). The EOM fits have been plotted over the data. (**e**) Stereoscopic image illustrating the size of an rMBP conformer and a DPC micelle of 54 detergent molecules^26^. (**f**) SANS EOM distributions of rMBP in the absence and presence of DPC micelles (solid and dashed lines, respectively). In the presence of DPC micelles (grey sphere), a distinct compacted population dominates (magenta cartoon) over the mixed elongated and compacted populations (black cartoons).

In the absence of additives, rMBP was always disordered in aqueous solution according to synchrotron radiation circular dichroism (SRCD) spectroscopy, which further revealed moderate helical folding in 2,2,2-trifluoroethanol (TFE) as well as with negatively charged sodium dodecyl sulphate (SDS) and several neutral detergent micelles (Supplementary Fig. S1). Using synchrotron small-angle X-ray scattering (SAXS), we found that rMBP was monomeric and highly elongated in solution (Fig. 1a, Supplementary Fig. S1, Supplementary Table S1). This explains the obtained radius of gyration (*R*_g_; 3.7–4.0 nm, depending on method) being higher than the measured *R*_h_; this is typical for particles deviating significantly from globularity^25^. The *ab initio* model (Fig. 1b) is elongated and similar to previously described models^20, 21^. Ensemble optimisation analysis (EOM) revealed distinct subpopulations of both *R*_g_ and maximum particle dimension (*D*_max_), suggesting the presence of different rMBP conformational species in solution (Fig. 1c). rMBP mostly adopts a reasonably compact conformation, close to that expected for a random polymer, instead of being fully extended (Fig. 1c, Supplementary Table S1). To study this further, we collected small-angle neutron scattering (SANS) data of rMBP in the absence and presence of *n-*dodecylphosphocholine (DPC) micelles (Fig. 1d, Supplementary Fig. S2, Supplementary Table S1). Size comparison of a computationally modelled DPC micelle^26^ and a single rMBP conformer suggests that each micelle can most likely only embed a single rMBP molecule (Fig. 1e), as suggested earlier^19^. It is noteworthy that the number of detergent molecules can vary within DPC micelles, with effects on micellar size and surface curvature^26^. Micellar scattering was masked using the contrast match-point of DPC, which allowed us to focus on the scattering of rMBP alone: EOM analysis of the SANS data revealed that DPC-bound rMBP shifted quantitatively to the more compact population, with a sharpened *R*_g_ distribution of 2.6–3.8 nm and a *D*_max_ distribution of 8–12 nm (Fig. 1f, Supplementary Table S1). Whilst being still relatively elongated, the data fit to a scenario where part of rMBP is embedded within a DPC micelle and partially folded, which is also supported by SRCD experiments (Supplementary Fig. S1) as well as SANS data analysis (Supplementary Fig. S2) using the Porod-Debye law^27^.

### Membrane interaction and folding of rMBP

**Figure 2:**
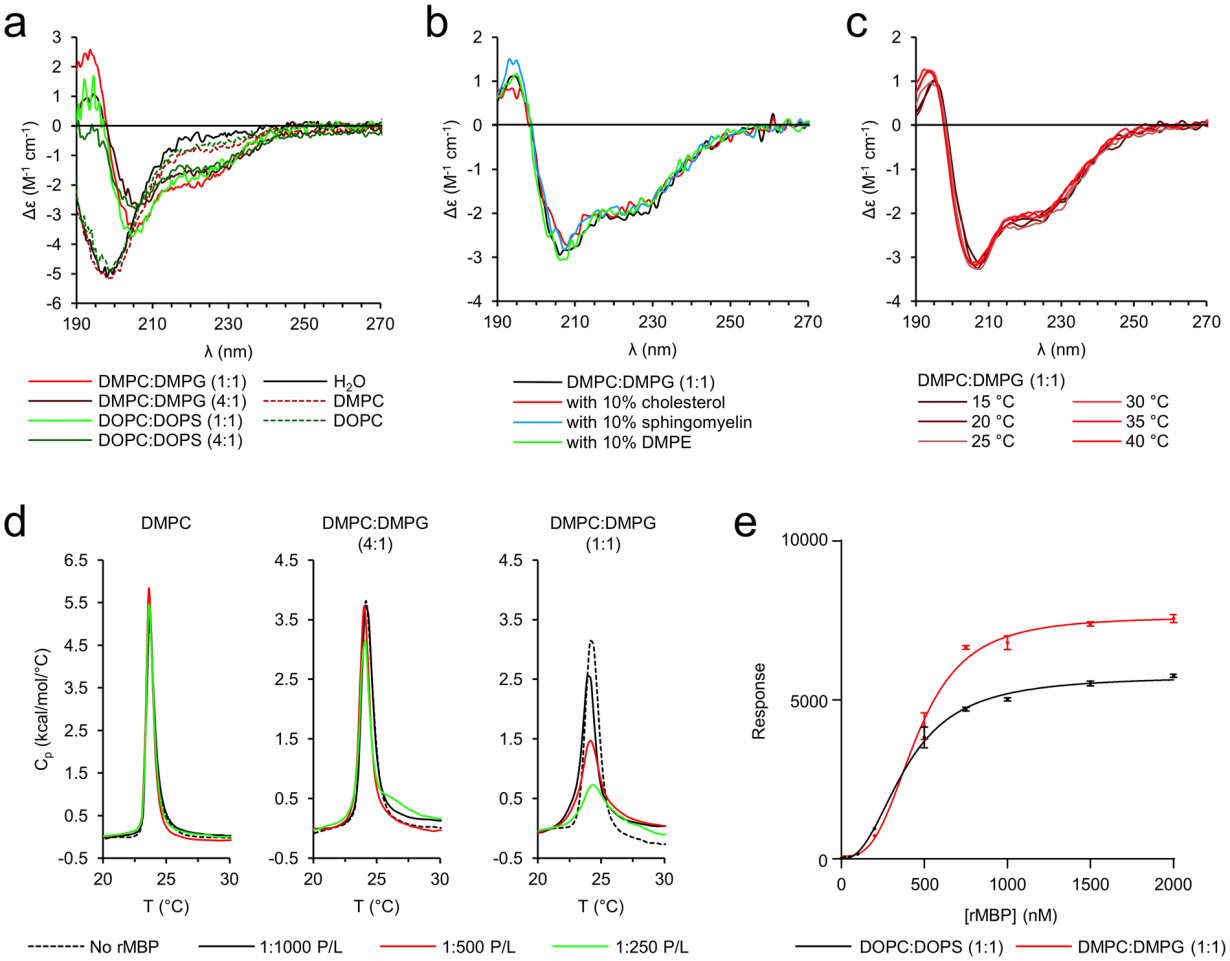
Lipid interactions of rMBP. (**a**) rMBP gains secondary structure in the presence of liposomes with increasing net negative surface charge, whereas it remains unfolded with net neutral lipids. (**b**) Cholesterol, sphingomyelin, or DMPE do not alter the secondary structure content of rMBP in net negatively charged DMPC:DMPG (1:1) vesicles. (**c**) Scanning around the lipid tail phase transition temperature does not affect the folding of MBP (right). (**d**) rMBP changes the lipid tail endothermic phase transition behaviour only when the MLV surface charge is net negative. (**e**) The association of rMBP with immobilised lipid vesicles probed using SPR. Error bars represent standard deviations.

We next investigated the membrane association of rMBP. For rapid screening of interactions, we used solution-state SRCD, as MBP gains secondary structure upon lipid binding^20^. rMBP reproducibly presented a clear increase in α-helical content with negatively charged small unilamellar vesicles (SUVs), with a slightly higher increase in the presence of saturated (dimyristoylphosphatidylcholine (DMPC): dimyristoylphosphatidylglycerol (DMPG)) than unsaturated lipids (dioleylphosphatidylcholine (DOPC): dioleylphosphatidylserine (DOPS)) (Fig. 2a). The importance of membrane surface charge is very clear, as rMBP does not undergo conformational changes with net neutral phosphatidylcholine lipids, indicating abolished, or at least altered or weakened, binding, as described previously^28^. To assess the effect of the abundant myelin lipids cholesterol, sphingomyelin, and phosphatidylethanolamine (DMPE) on folding, we included 10% (w/w) of each lipid separately in a standard DMPC:DMPG (1:1) mixture (Fig. 2b). None of these additives significantly affected the rMBP helical content, indicating that the surface net charge of the membrane dominates over lipid fluidity or hydrocarbon tails in initiating MBP folding.

The SRCD experiments provided evidence for membrane binding being mostly affected by electrostatics. Since the effect was practically the same with different lipid tails, we employed other strategies to observe possible membrane insertion. Differential scanning calorimetry (DSC) allows the determination of lipid phase transition temperatures, which reflect the conformational freedom of the lipid hydrocarbon tails. As a function of increasing temperature, dimyristoyl lipids present a small endothermic pre-transition (T_p_) from the planar gel phase (L_β_′) to the rippled gel phase (P_β_′), which is shortly followed by a major endothermic transition (T_m_) from P_β_′ to the liquid crystalline phase (L_α_) typically at +23–+24 °C^29^. We screened several protein-to-lipid (P/L) ratios of rMBP against DMPC:DMPG multilamellar vesicles (MLVs) with varying net negative surface charges using DSC, monitoring any rMBP-induced effects on the observed lipid T_m_ (Fig. 2d). Upon increasing the concentrations of rMBP, the endothermic main phase transition signal of negatively charged lipid membranes was broadened. Net neutral DMPC showed no difference in phase transition behaviour in the presence of rMBP. The data suggest MBP membrane insertion after charge neutralisation, supporting the conclusions from SRCD data.

DSC suggested lipid tail interactions with rMBP, even though the observed changes in rMBP conformation in SRCD with different lipid tails were minor. Next, we followed the conformation of rMBP in DMPC:DMPG (1:1) vesicles as a function of temperature (Fig. 2c). Scanning around the apparent T_m_ of dimyristoyl tails did not alter the conformation of rMBP, suggesting that lipid tail phase behaviour does not notably affect folding after rMBP has bound, but the dielectric environment inside the membrane is a maintaining factor for folding. To shed light on factors affecting binding affinity, we performed binding experiments of rMBP to immobilised DOPC:DOPS (1:1) and DMPC:DMPG (1:1) large unilamellar vesicles (LUVs) using surface plasmon resonance (SPR) (Fig. 2e). In a previous study^20^, we used SPR to address MBP-His binding to PC membranes in the presence of phosphoinositides, and we observed more binding with increased negative membrane charge, as well as a sigmoidal dependence of binding on MBP concentration. The association of rMBP with lipid vesicles was irreversible, as reported before for MBP-His^20^, since we observed essentially no rMBP dissociation from the lipids (data not shown). This suggests that the conformational change from disordered to helical structure occurs after initial electrostatic binding and anchors rMBP tightly onto the lipid surface. We performed saturation experiments by rMBP titration, which allowed the acquisition of key parameters of membrane binding (Fig. 2e, Table 1). While dimyristoyl-based lipids generally had higher responses (*R*_*hi*_) upon rMBP injection, the obtained saturation midpoint concentrations (*A*_1_), which can be considered apparent dissociation constants (*K*_*d*_), were similar for both DOPC:DOPS (1:1) and DMPC:DMPG (1:1), sharing the same surface net charge (Table 1). We performed a kinetic analysis by fitting the association phases of rMBP binding into a one-phase exponential association model (Supplementary Fig. S3). While the calculated *k*_on_ values are similar for both lipid compositions (Supplementary Table S2), the one-phase association model does not fit our data well enough to draw solid conclusions regarding association kinetics, and therefore, we conclude that MBP binding to a membrane surface is a complex binding event. It is clear that the initial association of rMBP onto a membrane surface is mostly governed by surface electrostatics, but the lipid tails do influence the amount of rMBP that can be integrated into liposomal bilayers, with a minimal effect on binding affinity or folding. Our results thus far clearly indicate rMBP membrane insertion and folding upon membrane binding.

**Table 1:** Surface plasmon resonance fitting parameters.

	R_hi_	R_lo_	A_1_	A_2_	R^2^
DOPC:DOPS (1:1)	5751 ± 79.84	−3.647 ± 48.78	389.5 ± 10.77	2.374 ± 0.1117	0.9978
DMPC:DMPG (1:1)	7624 ± 118.1	85.57 ± 73.49	455.5 ± 12.36	3.061 ± 02189	0.9968

### Effect of membrane fluidity and rMBP concentration on membrane stacking

**Figure 3:**
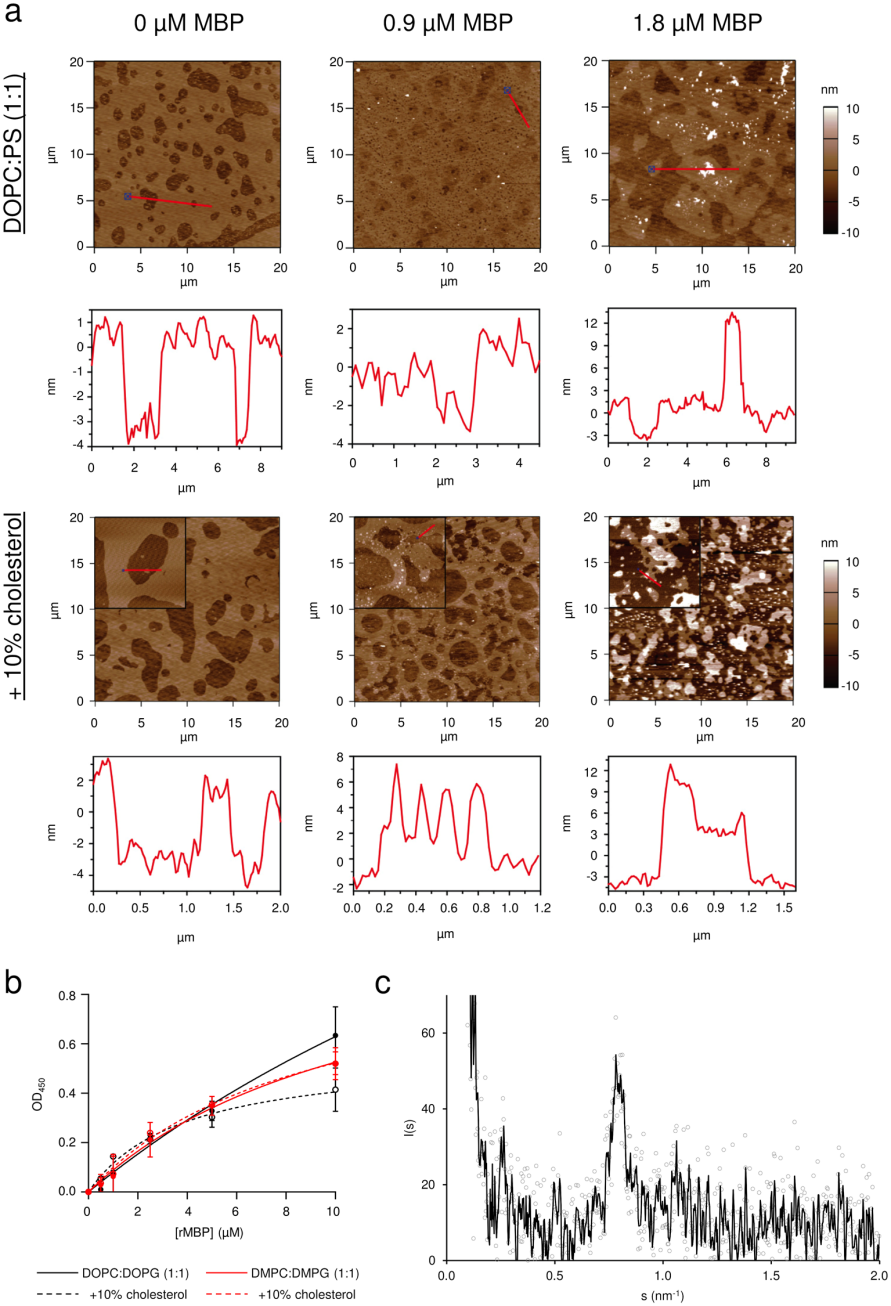
Membrane-stacking properties of rMBP. (**a**) In AFM, rMBP binds to supported DOPC:DOPS (1:1) lipid bilayers and spontaneously produces bilayer stacks after reaching sufficient protein concentration. The quality and area of the observed membrane stacks are influenced by the presence of cholesterol, without a significant effect on the measured stack thickness. Red lines denote the walk sections in the images, for which height diagrams have been plotted below. The walks from the cholesterol images have been extracted from 5-µm image insets for better quality. A single bilayer and stack are ~4 and 12 nm in height, respectively. (**b**) Turbidity measurements show that the tendency of rMBP to aggregate vesicles remains mostly unaffected by the lipid tail saturation degree or the presence of cholesterol. Error bars represent standard deviations. (**c**) rMBP mixed with DMPC:DMPG (1:1) SUVs displays a Bragg peak in SAXD that corresponds to a mean repeat distance of 80 Å at 1:100 molar P/L. A running average (black line) has been added for clarity over the raw measurement data (grey circles).

We have earlier shown spontaneous bilayer stacking by MBP and myelin protein P2 using AFM^14^. To observe this process in more detail, we prepared oriented DOPC:DOPS (1:1) bilayers with and without 10% (w/w) cholesterol on mica, and different concentrations of rMBP were added. After removal of unbound rMBP, the samples were imaged. rMBP bound onto the membrane surface and reproducibly induced membrane stacking, but the presence of cholesterol was not essential. However, the inclusion of cholesterol did cause the stacked membrane patches to appear larger and more uniform (Fig. 3a), suggesting that membrane fluidity plays a major role in stack formation, as demonstrated earlier using vesicle experiments^30^. We tested several concentrations of rMBP and found that a critical amount of rMBP has to accumulate onto the membranes before stacks emerge spontaneously: at 0.9 µM rMBP, membrane stacks were not typically observed, but at 1.8 µM, multilayers were always visible (Fig. 3a). Cholesterol did not affect this trend. In our earlier study, 1.8 µM MBP-His caused only minimal stacking of brain lipids, while extensive stacking was present at higher concentrations^14^. It is noteworthy that the observed critical concentration for membrane stacking in AFM corresponds to the rMBP concentration after reaching plateau for membrane surface binding in SPR. Thus, membrane stacking apparently requires surface saturation with MBP.

We next assessed the effect of lipid tail saturation and cholesterol on vesicle aggregation, similarly to our earlier experiments on P2^31^. The aggregation behaviour was the same for both DMPC:DMPG (1:1) and DOPC:DOPG (1:1), both with and without 10% (w/w) cholesterol (Fig. 3b), implying that the association of protein-decorated membranes is not dependent on cholesterol or lipid tail saturation. The differences in membrane fluidity resulting from cholesterol are most likely the reason to the observed stacking behaviour differences in AFM, as the presence of 10% (w/w) cholesterol did not notably affect the folding of rMBP either. Finally, we investigated whether rMBP produces vesicle aggregates with a distinct repeat distance. A Bragg peak corresponding to a mean repeat distance of 80 Å was present in our small-angle X-ray diffraction (SAXD) data from rMBP bound to DMPC:DMPG (1:1) vesicles (Fig. 3c). Bragg diffraction has been observed before from model vesicles and membranes as well as myelinated tissue^9, 15, 31, 32^. The result confirms the induction of ordered membrane stacks by rMBP, which were further used in imaging experiments.

### A membrane stack is uniformly occupied by a gel-like rMBP phase

**Figure 4:**
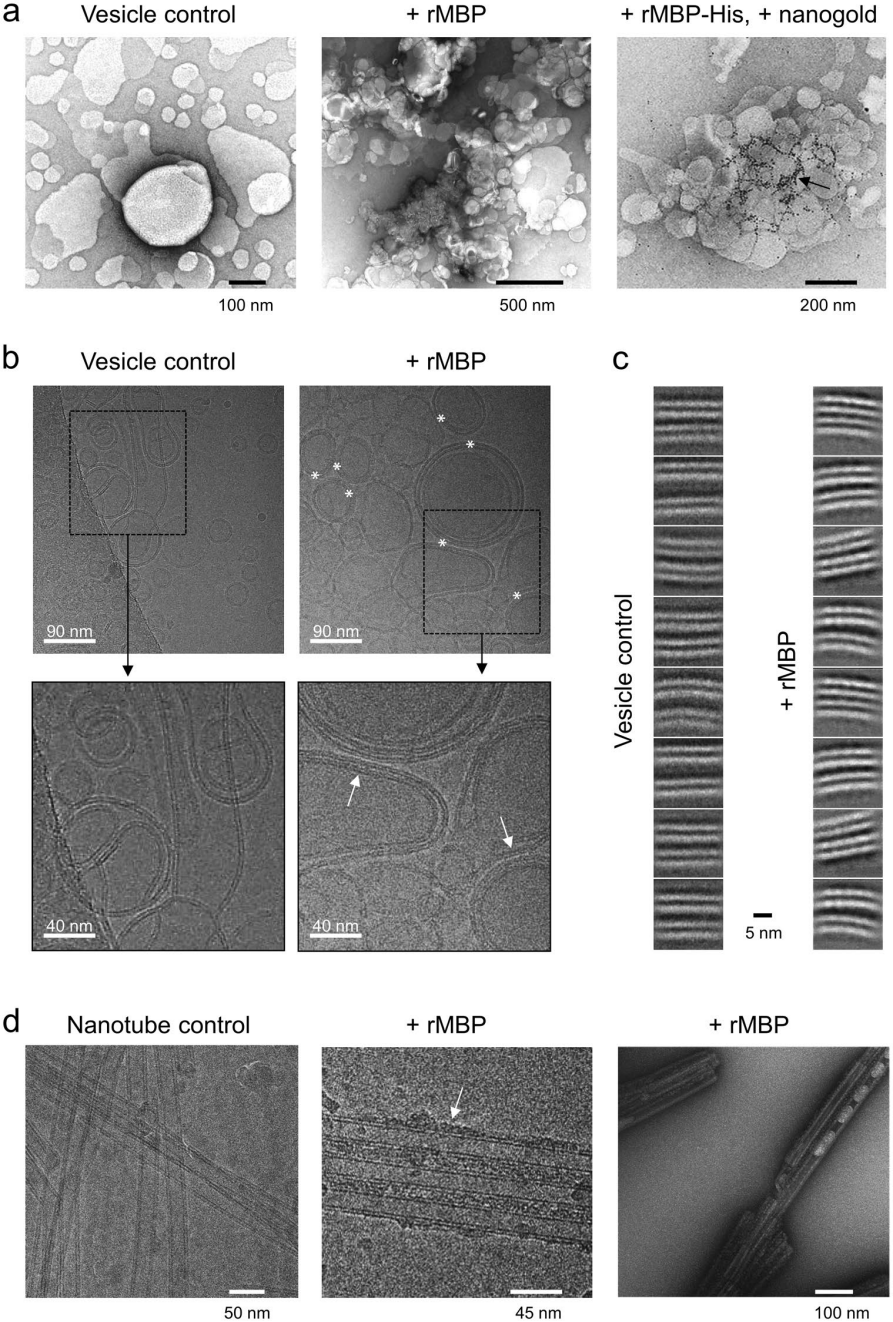
Architecture of rMBP in membrane stacks. (**a**) MBP aggregates vesicles and settles between membranes. rMBP-His was labelled with nanogold particles to localise the protein between vesicles (black arrow). (**b**) In cryo-EM, membrane stacking by MBP is evident when mixed with lipid vesicles (white asterisks). MBP also slightly flattens the membrane curvature of vesicles when more than one stack builds up (white arrows). (**c**) Particle analysis of cryo-EM imaged vesicles unveils an intermembrane compartment occupied by a uniform rMBP phase devoid of clear single particle boundaries, which brings the two apposing membranes into close vicinity to each other. The space between adjacent vesicle bilayers in the absence of rMBP is notably wider. (**d**) MBP coats GalCer-DOGS-NTA-Ni^2+^ nanotubes, displaying an extended conformation (white arrow). MBP also aggregates nanotubes, as evident from negatively stained samples (right).

To gain more detailed information about the assembly of MBP within membrane stacks, we studied MBP-aggregated vesicle samples using EM. As shown by nanogold labelling, in vesicle aggregates, MBP-His expectedly resided mostly between vesicle surfaces (Fig. 4a), as demonstrated earlier^33, 34^.

We investigated rMBP assembly between membranes using cryo-EM. We imaged rMBP-adhered vesicle membrane stacks and picked images of MBP between membranes for single particle analysis (Fig. 4b,c). rMBP settled tightly between the membranes and formed stacks with a total thickness of 11–13 nm (two 5-nm bilayers with a 1–3-nm protein phase between them). Particle analysis revealed that, as opposed to the membranes without rMBP that presented a loose space with a varying distance between apposing membranes, the intermembrane space in the presence of rMBP was occupied by a narrow uniform protein phase with no distinct globular particles. The space between lipid membranes was ~1 nm, with protein present both between and inside the membranes. Distances of ~2.5 nm between MBP molecules can be estimated, but the distances vary and the proteins are not clearly visible. This resembles the cohesive MBP meshwork proposed earlier^13^. Thus, further particle averaging and higher-resolution structure determination were not carried out. Despite the lipid bilayers in these experiments being symmetric, unlike in myelin, it is noteworthy that in cryo-EM, we only observed protein-containing stacks of two apposing lipid bilayers (Fig. 4b,c). This suggests that once MBP inserts into the membrane, it changes the membrane properties and can prevent further MBP insertion from the opposite side, unless a great excess of MBP is present. As shown by neutron scattering, the molecular dynamics of a lipid bilayer are affected by MBP ^15, 35, 36^. MBP can thus alter the overall structural and dynamical properties of an entire lipid bilayer, driving it towards asymmetric behaviour even when the lipid composition is symmetric. The molecular details of this asymmetry are currently unknown.

For observing concentration dependence of MBP-induced membrane stacking, we imaged vesicles with varying amounts of rMBP (Supplementary Fig. S4). While membrane multilayers were present in vesicle mixtures at high rMBP concentrations, stacking was lost as rMBP concentration decreased. Despite a different sample setup (freely tumbling vesicles) compared to AFM experiments (supported lipid bilayers), these observations do again suggest a critical MBP concentration for inducing membrane stacking.

We also imaged rMBP binding to lipidic nanotubes (Fig. 4d). rMBP bound nanotubes together, which was visible both with negatively stained samples as well as in cryo-EM. Compared to vesicles, nanotubes have different membrane fluidity, composition, and curvature. Importantly, we also observed the accumulation of rMBP onto nanotube exposed surfaces without stacking, corresponding to the accumulation of MBP onto a single membrane surface. As opposed to the more compact confinement within membrane stacks formed from vesicles, MBP appeared elongated when bound to nanotubes, similarly to DPC-bound rMBP in SANS.

### The formation of a protein phase on a single bilayer prior to stacking

The EM experiments raised a question regarding the conformation of rMBP on lipid bilayers prior to stacking. This is likely a physiologically relevant state during myelination; AFM demonstrated concentration dependence upon stack induction, and MBP can insert into membranes. Does MBP insert into a single membrane before a stack forms, and will MBP fold into a compact conformation before stacking? To answer these questions, we carried out NR experiments on uniform proteinous lipid bilayer samples.

The Langmuir-Blodgett/Schaefer approach to depositing lipid bilayers on solid substrates involves the sequential transfer of lipids from a Langmuir monolayer, with controlled area per molecule, by first slowly pulling the substrate out through the monolayer, with the interface perpendicular to the water surface (Langmuir-Blodgett) and then rotating the substrate by 90°, so that the interface is parallel to the water surface, and slowly pushing the substrate through the monolayer to deposit the outer leaflet of the bilayer (Langmuir-Schaefer). This approach allows precise control of the composition and coverage of both leaflets of the lipid bilayer independently, ensuring a high-coverage bilayer across the entirety of the large substrate surfaces necessary for NR measurements. NR is an excellent tool for studying *in-situ* the structure, perpendicular to the surface, of buried, hydrated thin films of biological and soft-matter molecules at the solid–liquid interface. The large penetrability of neutrons, combined with their high sensitivity to hydrogenated and deuterated materials, makes NR an ideal probe of interactions between proteins and biomembranes, simultaneously providing information on the interaction both at the surface and buried within the membrane.

**Figure 5:**
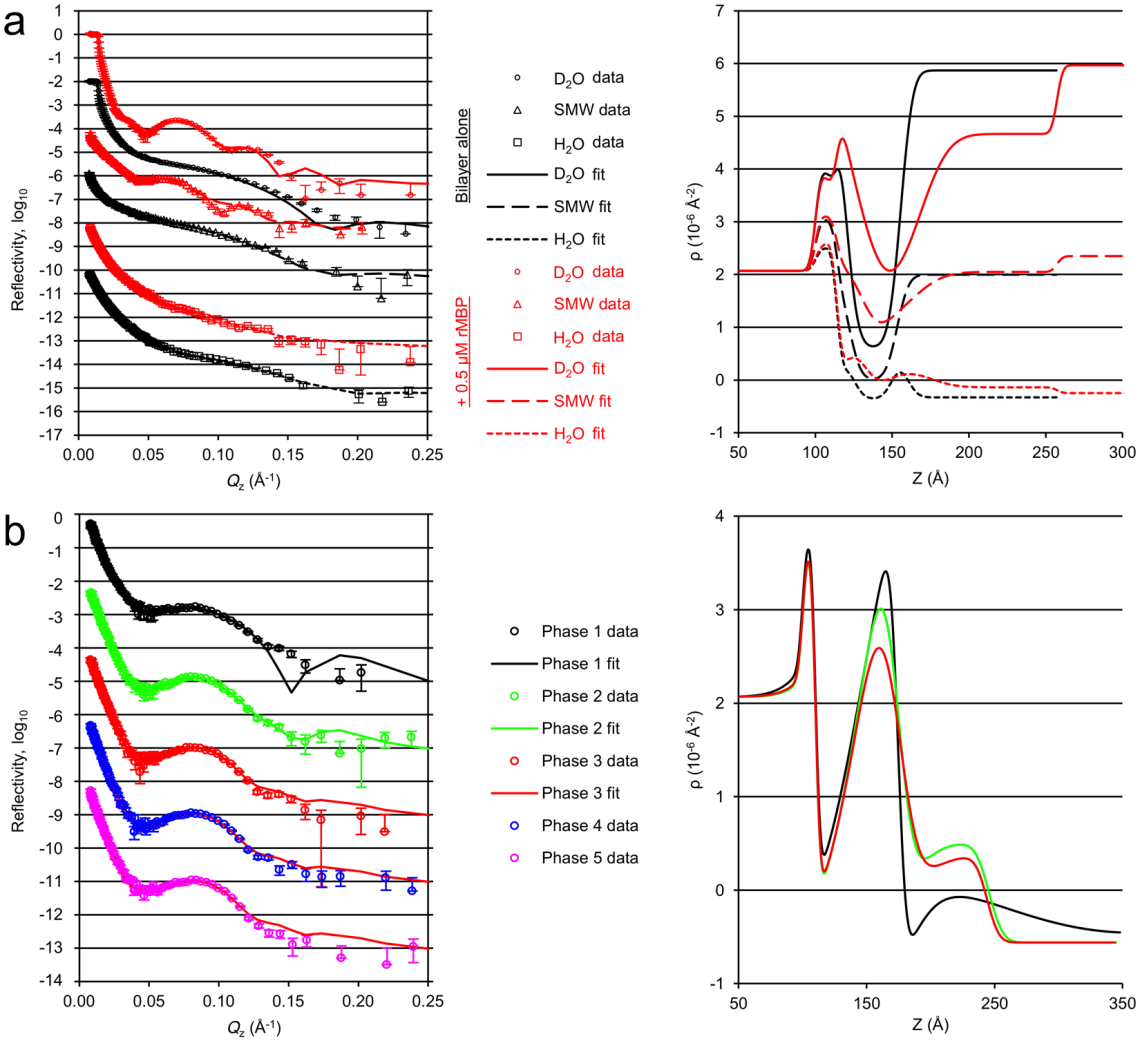
Accumulation of rMBP onto lipid bilayers. Data and fits are plotted on the left and scattering length density (ρ) profiles on the right. All reflectivity curves have been offset for clarity. (**a**) Steady-state NR experiment of DMPC:DMPG lipid bilayers with and without rMBP at different solvent contrasts. (**b**) Time-resolved NR experiment. The phases represent 15-min time-slices of rMBP associating with a d_54_-DMPC:d_54_-DMPG bilayer in an H_2_O contrast. The fitting curve for phase 3 has also been overlaid with phases 4 and 5, demonstrating steady state. The error bars represent standard deviations.

Using the Langmuir-Blodgett/Schaefer technique, we prepared uniform lipid bilayers of hydrogenated and perdeuterated DMPC:DMPG (1:1) on Si-crystal substrates with water as a subphase solvent. A flow cell setup allowed us to exchange the bulk solvent to buffers and to characterize the membranes with different solvent contrasts, as well as to inject rMBP inside the cell and follow its association as a function of time (Fig. 5a,b). Based on SPR and AFM, we chose to add rMBP onto the membrane at 0.5 µM, which retained the integrity of the membrane and did not induce spontaneous stacking (Fig. 5a). Initially, we observed that after injecting the protein, there was a time window that presented a rearrangement within the sample (Fig. 5b). After stabilisation of the sample and washing out unbound protein, the resulting reflectivity curve was best fitted to a model with a uniform protein layer, having a defined thickness and low roughness, on top of the existing DMPC:DMPG (1:1) bilayer, with full insertion of the protein into the outer leaflet of the bilayer, potentially displacing both lipid and water molecules from this leaflet (Fig. 5a, Table 2). Similar behaviour was observed with both hydrogenated and perdeuterated lipids (Fig. 5a, Supplementary Fig. S5), but not with DMPC alone (Supplementary Fig. S5), again demonstrating the importance of membrane surface charge. The parameters used to describe the best fit to each set of data clearly show that the protein layer covered ~25% of the membrane surface and was 7.5–8.5 nm thick. An additional ~3 nm of the protein was inserted within the outer leaflet of the bilayer, displacing ~40% of the lipids in this leaflet and leading to a rearrangement of the membrane to support this. The analysis suggests compaction compared to the most elongated conformers suggested by SAXS, fitting well to the narrowed EOM population observed by SANS for DPC-bound rMBP. The protein layer, however, does not represent a completely collapsed MBP, thought to reside within the mature bilayer stack^5^, but a previously undetected pre-stack MBP conformation: a brush-like layer of mostly disordered protein, anchored onto the membrane most likely *via* one or more helical segments. The introduction of another membrane leaflet could result in a second insertion event, followed by protein folding and compaction, a single MBP molecule potentially penetrating both bilayer surfaces. The protein meshwork has been suggested to be composed of laterally self-assembled^12^ or dimeric MBP^12^. Taking into account our observations and earlier data, it is obvious that MBP forms a protein phase onto a membrane surface, and this process involves also self-association. However, we find it unlikely that MBP would, even when bound to membranes within compact myelin, fold into structurally highly ordered assemblies with a well-defined oligomeric state.

**Table 2:** Neutron reflectometry parameters.

Parameters		DMPC:DMPG (1:1)		d_54_-DMPC:d_54_-DMPG (1:1)	
		Bilayer alone	With rMBP	Bilayer alone	With rMBP
Substrate	Oxide thickness (Å)	14 ± 1		10 ± 1	
	Oxide coverage (%)	75 ± 1		90 ± 4	
	Oxide roughness (Å)	3 ± 1		3 ± 2	
	Hydration layer between oxide and bilayer (Å)	5 ± 1	5 ± 2	20 ± 5	25 ± 6
Bilayer	Bilayer area-per-molecule (Å^2^/molecule)	60 ± 4	45 ± 3	63 ± 8	52 ± 10
	Water molecules per lipid head	3 ± 1	10 ± 2	3 ± 1	10 ± 1
	Water molecules per lipid tail	4 ± 2	17 ± 3	6 ± 2	17 ± 2
	Global bilayer roughness (Å)	5 ± 1	8 ± 1	18 ± 6	22 ± 3
	Local bilayer inner roughness (Å)	3 ± 1	4 ± 2	3 ± 2	5 ± 1
	Local bilayer outer roughness (Å)		11 ± 2		0 ± 3
rMBP	MBP in outer leaflet (%)		39 ± 7		44 ± 9
	MBP layer thickness (Å)		84 ± 2		75 ± 4
	MBP layer coverage (%)		26 ± 1		23 ± 3
	MBP layer roughness (Å)		3 ± 2		10 ± 4

To further study the protein-membrane association, we added 0.5 µM rMBP to a membrane in a time-resolved NR experiment (Fig. 5b, Supplementary Table S3). While this concentration is not high enough to provide information about stacking, we could observe the entire association process of rMBP. It should be noted that this experiment was carried out with a single contrast, increasing the uncertainty in the fitted parameters. However, clear trends were still seen, as the association took place on a reasonably long time scale, during which three distinct phases could be resolved: initially a 7-nm thick, diffuse protein layer formed on the membrane, with a very high roughness, indicating high heterogeneity and no insertion into the membrane. Next, this layer collapsed into a denser, more homogeneous layer with some insertion into the headgroup region of the bilayer outer leaflet. Only after this, one can observe full membrane insertion, displacing lipids from the outer leaflet of the bilayer, which suggests that rMBP has to accumulate and fold onto the membrane prior to embedding. The coverage of the protein was very high during these time-resolved measurements, up to 50%, as opposed to the buffer-exchanged sample (~25%; Table 2), with less displacement of lipid from the outer leaflet, indicating that the rinsing of the membrane may remove some additional lipid molecules along with excess protein. The third phase in our time-resolved measurement was essentially the steady state, and the system remained stable beyond this time point (phases 4 & 5 in Fig. 5b).

## Discussion

Using a recombinant form of MBP accurately mimicking the major 18.5-kDa isoform, we have provided a full physicochemical picture of MBP-induced stacking of lipid bilayers. MBP is an intrinsically disordered protein in solution, which after lipid membrane binding gains secondary structure and embeds deep into the bilayer. On top of the membrane, an amorphous protein brush is formed, which is likely to be of high importance, when an apposing membrane is bound. A fully compacted MBP conformation is only present between two bilayers. Our results provide a comprehensive molecular framework for membrane binding and stacking by MBP, which is a peripheral membrane protein with unique molecular properties.

Our extensive characterisation of tag-free rMBP shows that MBP is disordered in solution and binds lipid membranes through electrostatic interactions. While the lipid composition of myelin is more complex than that used in the current study, we decided here to focus on simple lipid mixtures that generally behave well in various biophysical techniques and can be used to screen for basic membrane properties, including surface charge, lipid headgroup, level of saturation, and fluidity. While the lipid hydrocarbon tails do not affect the binding affinity, kinetics, or folding of MBP substantially, the dynamic freedom and spatial occupation of the tails define how much MBP can bind to saturate the membrane surface. MBP also affects the biophysical properties of the entire lipid bilayer, as for example highlighted by the observation of MBP-induced stacks of only two, but not more, bilayers in cryo-EM, as well as previous studies with cultured oligodendrocytes^37, 38^. The importance of both the lipid and protein components in affecting the dynamics and function of each other has been highlighted earlier for both MBP^17, 35, 36, 39^ and myelin protein P2^40, 41^.

**Figure 6:**
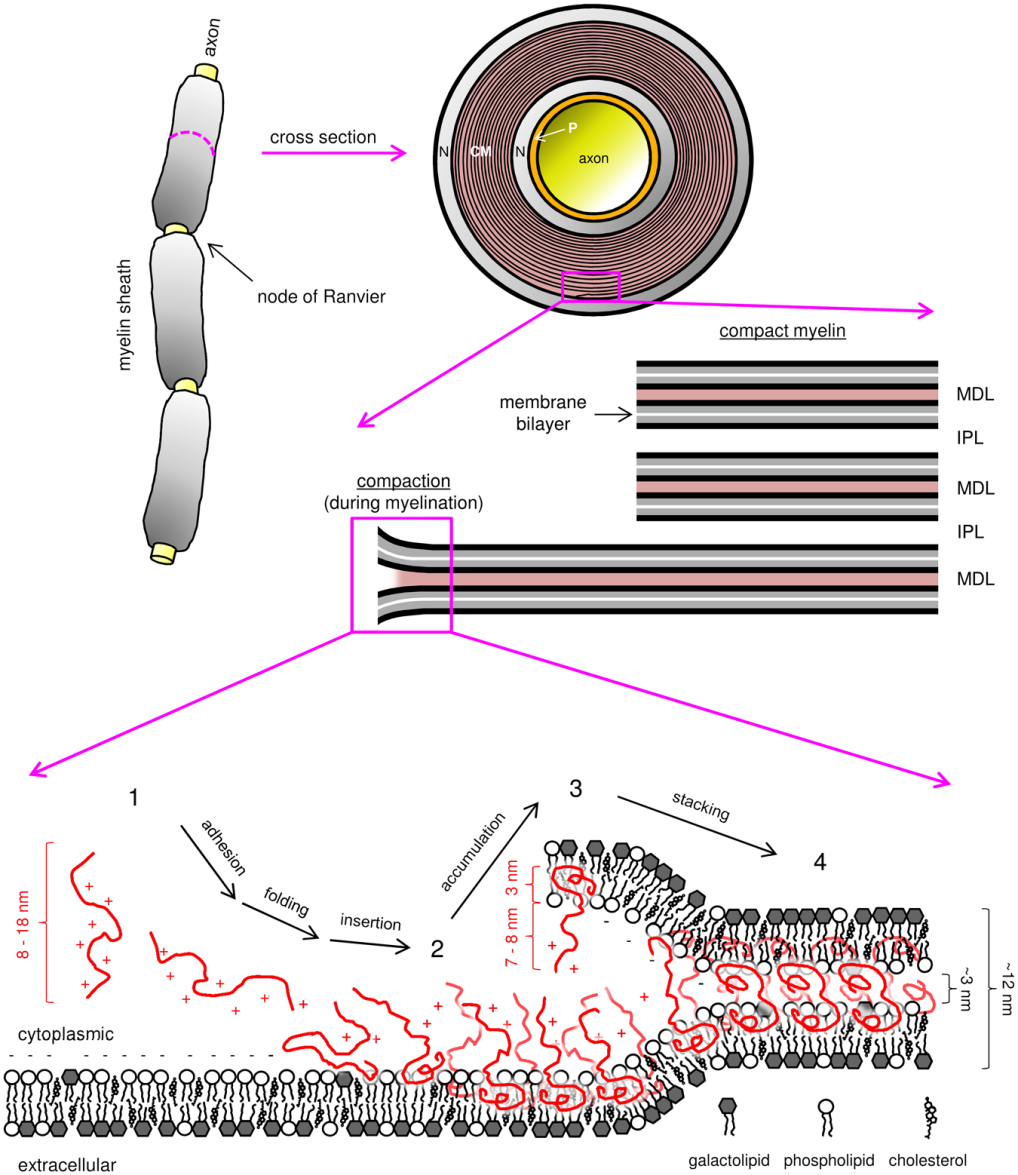
The association landscape of MBP with myelin membranes leads to the formation of MDL. MBP (red) in solution is disordered and approaches the cytoplasmic membrane surface through electrostatic interactions (1), which eventually leads to charge neutralisation, subsequent folding, and membrane insertion (2). Membrane-adhered MBP accumulates on the surface, which increases the binding propensity of a second membrane leaflet (3). Once a critical surface occupancy is reached, another insertion and possible folding event occurs, resulting in a dense protein phase that forms the 3-nm MDL within the 12-nm membrane stack (4). CM, compact myelin; N, non-compact myelin; P, periaxonal space; MDL, major dense line; IPL, intraperiod line.

Our results demonstrate that charge neutralisation is a key event before MBP folding and membrane insertion can take place. Once charge neutralisation and membrane saturation with MBP occur, a string of downstream events is triggered, ultimately leading to stable myelin-like membrane stacks. Based on our observations, and considering earlier literature, we now propose a molecular mechanism for the stepwise, MBP-driven formation of the MDL (Fig. 6).

In the initial step, the highly positively charged, disordered MBP is attracted to a negatively charged membrane surface by electrostatic interactions, which starts the association cascade. When encountering the membrane surface, MBP binds through electrostatics, and opposite charges are neutralised – this allows other factors, such as hydrogen bonding and the hydrophobic effect, to take over and causes MBP to fold and subsequently insert deeper into the inner membrane leaflet, and even partially into the outer, as proposed already decades ago^30^. The process occurs in a fairly small volume: only partial protein insertion and folding occurs, while most of MBP remains elongated outside the membrane, maintaining a positive net charge. This is plausible, as several distinct segments of MBP have been shown to have propensity to fold independently upon interacting with lipids/detergents^42–46^. This initial association, however, may already be essentially irreversible^12, 20, 42^, although it is likely that there are folded species in the system that present weak affinity and are removable, possibly due to electrostatic repulsion.

Next, given the fairly confined electrostatic field of the lipid bilayer, which gets neutralised by the embedded protein, more MBP continues to bind and integrate within the single membrane leaflet. A fairly thick amorphous peripheral protein phase emerges, which translates into a strong accumulation of positive charge covering the membrane surface. This protein layer is likely to represent the distinct MBP protein phase described earlier^13^. Brush-like phases consisting of disordered proteins and peptides have been recently reported^47, 48^. Our SANS data in the presence of detergent micelles provide evidence for a brush-like property for membrane-bound MBP, although the detailed organisation of this protein phase currently remains unknown. This phase was also visualised by cryo-EM on the surface of lipid nanotubes, providing a starting point for further studies into its molecular details.

Finally, when a critical surface-bound MBP concentration has been reached, the soft protein brush adheres to a second membrane leaflet, resulting in full charge neutralisation. MBP undergoes a second compaction event to fit into the confined space between the two bilayers, forming a meshwork of extraordinary stability between the two cytoplasmic membrane leaflets. It is likely that this phase transition is governed by specific aromatic residues in MBP^13^. The meshwork may involve lateral MBP assembly^9, 12^, and the 3-nm MDL can easily be spanned by a single MBP monomer, as suggested earlier^5, 16, 17, 49^. During myelination, both membrane bilayers involved in stack formation are likely to accumulate MBP continuously. Since the membranes are in close proximity to each other, the stacking could proceed *via* a zipper-like mechanism, whereby only the leading part of accumulated MBP reaches the critical concentration at a given time and effectively transitions into a molecular adhesive. Interactions of MBP with cytosolic proteins, such as calmodulin^20, 50^ or the cytoskeleton^19, 51^, may regulate this process.

The above string of events is a feasible molecular model for myelin membrane stacking, assuming enough MBP is available locally. During CNS myelination, MBP is translated in the vicinity of the growing membrane from transported mRNA^52^. The local synthesis of MBP is likely to be a key triggering factor for myelin membrane compaction. In myelin formation and maintenance, cytoskeletal networks and other proteins, such as 2′,3′-cyclic nucleotide 3′-phosphodiesterase (CNPase), play central roles^53^. An interplay between MBP and membrane-bound CNPase may be important, as both CNPase and MBP bind microtubules and actin^19, 51^. Recent data also suggest that myelin formation is driven by cytoskeletal disassembly^54^, in addition to proteolipid protein-driven myelin formation^55–57^. CNPase overexpression inhibits MBP-based myelin compaction and causes aberrations in the periaxonal membrane^58^. A model outlining the interplay between CNPase and MBP in maintaining the interface and balance between CM and cytosolic channels was recently proposed, showing antagonistic effects of CNPase and MBP on channel formation^59^.

Changes in the expression patterns of key proteins (*e*.*g*. CNPase) during myelination and in mature myelin could switch the formation and stability of the MDL to a point where MBP is re-exposed to the cytosol, making it susceptible to proteolysis. While we do not yet know enough about the reversibility of myelination at the molecular level, MBP accumulated on a single membrane surface is a more suitable substrate for proteases and other modifying enzymes than a folded, membrane-embedded MBP. This could further decrease the stability of the MDL through disruption of the MBP meshwork, setting out a rolling wheel that may eventually result in a neurological condition, possibly by exposing autoimmune epitopes of MBP and other myelin proteins. Autoantigenic MBP peptides, as well as molecular mimics thereof, have been discovered and characterised^60^. These could be disease-triggering or even preventive factors when modified correctly^61, 62^, the underlying mechanisms currently being unclear. Picturing the above molecular steps of MDL formation should allow to better understand the myelination process as well as demyelinating disorders.

Stability of the proteolipid membrane multilayer is a key factor for keeping myelin healthy. Initial interactions between MBP and lipid membranes are mostly electrostatic, and deiminated charge variants of MBP have been linked to MS^4^. Decreased positive charge on MBP leads to weaker membrane interactions^20, 42, 63^. Modified variants could affect myelin stability through slight structural, functional, and compositional differences, as well as susceptibility to proteolysis^10, 11, 20^. The concentration of divalent ions, most importantly Ca^2+^ and Zn^2+^, is likely to influence the formation and stability of compact myelin^64^. An obvious factor to consider is the membrane lipid composition; the negatively charged cytoplasmic leaflet and the high cholesterol content of myelin are most probably in a delicate balance with the rest of the different lipid species, as shown by experimental disease models^9, 49, 65^. The high abundance of cholesterol has a major impact on membrane fluidity as well as myelin development^30, 66, 67^, likely being a key requirement together with various phosphoinositides^68–70^ to provide a suitable physiological medium for membrane-embedded proteins, including MBP. The stability of the MDL, the condition of MBP within it, and the post-natal myelinating machinery should be taken into account when it comes to understanding and possibly treating demyelinating diseases.

To conclude, through the use of multidisciplinary methodologies, the structural importance of MBP as a multifunctional compact myelin protein is unfolding. An MBP-driven scheme outlining the formation and stability of the myelin MDL unravels, while the MDL remains a poorly understood compartment of developmental, functional, and medical importance. The molecular steps of formation of the myelin MDL rely on an equilibrium between electrostatic and hydrophobic interactions, protein concentration, membrane association, and charge accumulation to form the final product: a stable, dense protein phase that occupies the MDL and binds lipid bilayers tightly together. The elucidation of the molecular foundation of MBP-mediated membrane stacking allows us to better understand this intriguing biological system, and it could act as a key to unlock the discovery of remedies to conditions that present aberrant myelination and disturbed myelin ultrastructure. To further refine the model, more complex molecular systems should be studied, such as multiprotein systems including integral membrane proteins of myelin. Other myelin proteins, *e*.*g*. the cytoplasmic extension of myelin protein zero^71^ and P2^14, 15, 31^, function similarly to MBP, likely leading to functional synergies and possibly competition. Once the underlying detailed kinetics and the effects of other molecular components of myelin, including proteins and ionic species, on proteolipid membrane association and stacking are deciphered, we can truly understand the complexity of myelination in a logical, structured manner.

## Methods

### Cloning, expression, and purification

A synthetic gene encoding the 18.5-kDa isoform of mouse MBP (DNA2.0) was subcloned into the Gateway donor vector pDONR221 (Life Technologies). A Tobacco Etch Virus (TEV) protease digestion site (ENLYFQG) was added before the gene, and *att*B1 and *att*B2 recombination sites before and after the gene, respectively. This entry clone was used to generate an expression clone in the pTH27 vector^72^, which codes for an N-terminally His_6_-tagged protein (His-rMBP).

The expression of His-rMBP was performed in the *E*. *coli* BL21(DE3)pLysS RARE strain in LB medium at +37 °C using a 2-h induction with 1 mM isopropyl β-D-1-thiogalactopyranoside, after an initial culture OD_600_ of 0.3 had been reached. After harvesting, cell pellets were re-suspended into 50 mM HEPES (pH 7.5), 500 mM NaCl, 6 M urea, 20 mM imidazole, 1 mM phenylmethylsulphonyl fluoride, with added EDTA-free protease inhibitors (Roche), and lysed by ultrasonication. His-rMBP was purified using Ni-NTA chromatography. Elution was done using 40 mM HEPES (pH 7.5), 400 mM NaCl, 4.8 M urea, 500 mM imidazole, and the elution fraction was dialysed sequentially against several dialysis reservoirs, lowering the urea content in a stepwise manner from 4.8 to 2 M. After this, recombinant TEV protease was added to digest the N-terminal His_6_-tag of His-rMBP, resulting in a near-native 18.5-kDa rMBP with one extra N-terminal Gly residue from the TEV digestion site. Cleavage was carried out overnight at +4 °C, while dialyzing against 40 mM HEPES (pH 7.5), 400 mM NaCl, 1 M urea. The digested protein was dialysed back into 50 mM HEPES (pH 7.5), 500 mM NaCl, 6 M urea, 20 mM imidazole, and another Ni-NTA purification was performed. The unbound and wash fractions were combined and dialysed against 40 mM HEPES (pH 7.5), 400 mM NaCl overnight. After the final dialysis step, protein was concentrated and subjected SEC on a Superdex 75 pg column (GE Healthcare). Either HBS (10 mM HEPES, 150 mM NaCl, pH 7.5) or 20 mM HEPES (pH 7.5), 300 mM NaCl, 1% (w/v) glycerol was used as the running buffer, depending on downstream experiments. The purity of the protein was checked using SDS-PAGE and DLS using a Malvern Zetasizer Nano ZS instrument. The SEC fractions containing pure rMBP were snap-frozen using liquid N_2_ and stored at −80 °C. The protein fractions were thawed, pooled, and concentrated immediately before downstream experiments.

C-terminally His-tagged MBP (MBP-His^20^) was expressed and purified similarly to rMBP, omitting TEV digestion and the second Ni-NTA step. After the first Ni-NTA step and stepwise dialysis, the protein was immediately subjected to SEC, and the fractions containing pure MBP-His were snap-frozen.

### Mass spectrometry

The accurate molecular mass of rMBP was determined using liquid chromatography-coupled electrospray ionisation time-of-flight mass spectrometry in positive ion mode, using a Waters Acquity UPLC-coupled Synapt G2 mass analyser with a Z-Spray ESI source. The identity of rMBP was further verified using peptide fingerprinting by in-gel trypsin proteolysis and matrix-assisted laser desorption/ionisation time-of-flight mass spectrometry with a Bruker Ultra fleXtreme mass analyser. The peptide fingerprints were compared directly against theoretical peptides from the protein sequence.

### Small-angle scattering

SAXS data for rMBP were collected from samples at 1–4 mg ml^−1^ on the EMBL P12 beamline, DESY (Hamburg, Germany). The buffer contained 20 mM HEPES (pH 7.5), 300 mM NaCl, and 1% (w/v) glycerol. Monomeric bovine serum albumin was used as a molecular weight standard. See Supplementary Table S1 for further details.

SANS data for 2.3 mg ml^−1^ (124 µM) MBP in the absence and presence of 0.25% (7.1 mM) DPC micelles were collected on the D22 beamline, ILL (Grenoble, France) using a 1-mm pathlength Hellma 100-QS quartz cuvette at +10 °C with a 1-h exposure time. The used buffer was 20 mM Tris-HCl (pH 7.5), 150 mM NaCl. The measurements were carried out at 4-m collimation and sample-detector distances, using a monochromatic neutron wavelength of 6 Å ± 10%. Data were corrected for the transmission, the pathlength, the empty cell, and the blocked beam, and scaled to absolute intensity using the measurement of beam flux at the sample position. Neutron scattering data for 5 mg ml^−1^ DPC micelles in 100, 80, 60, 40, 20, and 0% D_2_O were collected, and a contrast match point of DPC was determined to be 9% D_2_O, as previously described^73^. The used D_2_O solvent contrasts were 9% and 98% for MBP with and without DPC, respectively. Initial data processing was done using GRASP (www.ill.eu/instruments-support/instruments-groups/groups/lss/grasp/home/) and NCNR SANS reduction macros for Igor^74^.

Data were processed and analysed using the ATSAS package^75^. GNOM was used to calculate distance distribution functions^76^, and *ab initio* modelling was performed using GASBOR^77^. Ensemble optimisation analysis was performed using EOM^78^.

### Liposome preparation

Cholesterol, DMPC, DMPE, DMPG, dioleylphosphatidylglycerol (DOPG), DOPC, and sphingomyelin were from Larodan Fine Chemicals AB (Malmö, Sweden). DOPS and the deuterated d_54_-DMPC and d_54_-DMPG were from Avanti Polar Lipids (Alabaster, Alabama, USA).

Lipid stocks were prepared by dissolving dry lipids in chloroform or chloroform:methanol (1:1 v/v) at 5–10 mg ml^−1^. Mixtures were prepared from the stocks at desired ratios, followed by solvent evaporation under a gentle stream of N_2_ and freeze-drying for at least 4 h at −52 °C under vacuum. The dried lipids were either stored air-tight at −20 °C or used directly to prepare liposomes.

Liposomes were prepared by adding either deionised water or HBS to the dried lipids to reach a final concentration of 2–10 mg ml^−1^, followed by vigorous mixing and gentle sonication for 15 min in a water bath sonicator at ambient temperature, to ensure that no unsuspended lipids remained in the vessel. MLVs were prepared by seven cycles of freeze-thawing using liquid N_2_ and a warm water bath, with vigorous vortexing after each cycle. LUVs were prepared by extruding MLVs 11 times through a 0.1-µm membrane on a +40 °C heat block and used immediately in experiments. SUVs were prepared using sonication of MLVs. Either probe tip sonicators (a Branson Model 450 and a Sonics & Materials Inc. Vibra-Cell VC-130) or a strong water bath sonicator with temperature control (UTR200, Hielscher, Germany) were used to clarify the liposome suspensions, while avoiding overheating. The SUVs were immediately used in experiments.

### Synchrotron radiation circular dichroism spectroscopy

SRCD data were collected from 0.3–0.5 mg ml^−1^ protein samples in water on the UV-CD12 beamline at ANKA (KIT, Karlsruhe, Germany)^79^ and the AU-CD beamline at ASTRID2 (ISA, Aarhus, Denmark). Samples with lipids were prepared on-site by mixing rMBP and freshly sonicated SUVs, followed by 5–10 min of degassing using a water bath sonicator at ambient temperature. 100-µm pathlength closed circular cells (Suprasil, Hellma Analytics) were used. SRCD spectra were measured from 170 to 280 nm at +30 °C, and the raw CD units were converted to Δε (M^−1^ cm^−1^), using rMBP concentration determined from absorbance at 280 nm. SDS and TFE were purchased from Sigma-Aldrich and the detergents LDAO, OG, and DPC from Affymetrix. The unfolded nature of rMBP, as well as its tendency to fold in DMPC:DMPG (1:1) and DOPC:DOPS (1:1), were reproducible between beamtime sessions.

### Differential scanning calorimetry

Various concentrations of rMBP were mixed with MLVs in HBS containing 160 µM of either DMPC, DMPC:DMPG (4:1), or DMPC:DMPG (1:1), in a final volume of 700 µl. Lipid samples without added rMBP were prepared as controls. The P/L ratio was 1:250–1:1000. The samples were incubated at +37 °C for 10 min to ensure thorough protein association with the vesicles, and degassed in a vacuum with stirring at +10 °C prior to measurements. All samples were prepared and measured in duplicate, the observed trends being reproducible.

DSC was performed using a MicroCal VP-DSC with a cell volume of 500 µl. A HBS reference was used for all samples. Each calorimetric cycle (one per sample) was performed from +10 to +50 to +10 °C with 1 °C min^−1^ increments. Baselines were corrected and zeroed at +20 °C to make cross-comparison straightforward.

### Surface plasmon resonance

SPR was performed on a Biacore T200 system (GE Healthcare). According to the manufacturer’s protocol, 100-nm LUVs of 1 mM DMPC:DMPG (1:1) and 1 mM DOPC:DOPS (1:1) were immobilised on separate channels on an L1 sensor chip (GE Healthcare) in 20 mM HEPES (pH 7.5), 150 mM NaCl, followed by the injection of rMBP. Chip regeneration was done with a 2:3 (v:v) mixture of 2-propanol and 50 mM NaOH. rMBP was at 20–2000 nM in the running buffer, and a single concentration per each lipid capture was studied; all samples were performed in duplicate, with one sample in each series measured twice to rule out instrumental artifacts or deviations. The binding response as a function of protein concentration was plotted and fitted to the 4-parameter model

1 $$R={R}_{hi}-\frac{{R}_{hi}-{R}_{lo}}{1+{(\frac{[MBP]}{{A}_{1}})}^{{A}_{2}}},$$

to gain information about association affinity. For kinetic analyses, all association phases (180 s after injection of rMBP) were individually fitted to a one-phase exponential association model using GraphPad Prism 7. The obtained *k*_obs_ values were plotted against rMBP concentration and fitted using linear regression to determine *k*_on_ (slope of the curve) and *k*_off_ (Y-intercept of the curve). The latter values were extracted from two individually fitted datasets: one containing all data (Fitting set 1) as well as one omitting data points below 500 nM rMBP (Fitting set 2).

### Vesicle aggregation and small-angle X-ray diffraction

SUVs of 0.5 mM DOPC:DOPG (1:1) and DMPC:DMPG (1:1), both with and without supplemented 10% (w/w) cholesterol, were mixed with 0.5–10 µM rMBP in duplicate. Turbidity was recorded at 450 nm for 10 min at +25 °C using a Tecan M1000Pro plate reader. The results were analysed after turbidity was stable.

SAXD was performed to find the mean repeat distance of rMBP-stacked multilayers in aggregated lipid vesicle samples. 2–20 µM MBP was mixed with SUVs of 1–3 mM DMPC:DMPG (1:1) in HBS at ambient temperature and analysed at +25 °C on the EMBL P12 BioSAXS beamline, DESY (Hamburg, Germany). Buffer references were subtracted from the data. Lipid samples without added rMBP were used to verify the absence of Bragg peaks. The peak positions of momentum transfer, *s*, in rMBP-lipid samples were used to calculate the mean repeat distance, *d*, in a proteolipid multilayer, using the equation

2 $$d=\frac{2\pi }{s},\,{\rm{where}}\,{\rm{s}}=\,\frac{4{\rm{\pi }}\,\sin \,{\rm{\theta }}}{{\rm{\lambda }}}.$$

### Atomic force microscopy

Fresh DOPC:DOPS (1:1) and DOPC:DOPS (1:1), 10% (w/w) cholesterol SUVs were unrolled on freshly cleaved mica (∅ 1.2 cm) in HBS-Ca (10 mM HEPES, 150 mM NaCl, 2 mM CaCl_2_, pH 7.5), by covering the mica entirely with 0.2 mg ml^−1^ SUVs, incubating for 20 min at +30 °C, and washing twice with HBS-Ca.

Freshly prepared samples were imaged in HBS at ambient temperature using an Asylum Research MFP-3D Bio instrument and TR800PSA cantilevers (Olympus; spring constant (*k*) range 0.59–0.68 N m^−1^, resonance frequency 77 kHz) in alternative current (AC) mode. Square 256 × 256 pixel scans were acquired from areas of 5–20 µm, with a 90° scanning angle and a scan speed of 0.6–0.8 Hz. The resulting scan images were processed with Igor Pro 6.37.

After confirming the presence of lipid bilayers, 0.5–3.6 µM MBP was added onto the bilayer samples in HBS. The samples were incubated for 15 min at ambient temperature, washed twice with HBS, and scanned as above. For every protein concentration, 2–6 samples were prepared and scanned with excellent reproducibility. Scans from at least 3 different areas for each sample were acquired to rule out artifacts originating from sample heterogeneity.

### Electron microscopy

#### Protein reconstitution into lipid vesicles

The lipid mixture contained polar brain lipid extract (Avanti), palmitoyloleoylphosphatidylcholine (POPC, Avanti), and cholesterol (Anatrace) in the ratio 3:1:1. Vesicles were prepared as above, except that organic solvent was evaporated under argon and the sample vacuum-dried at room temperature, prior to resuspending in aqueous solvents. For EM experiments, 0.4 mg ml^−1^ of rMBP was mixed with 0.4 mg ml^−1^ polar brain lipid:POPC:cholesterol vesicles in a buffer containing 20 mM Tris pH 7.5, 200 mM NaCl, 1% glycerol, and the sample was incubated for 24 h at room temperature. For negatively stained EM imaging of lipid vesicles, samples containing 730 µM DMPC:DMPG (1:1) SUVs were prepared with 0–7.3 µM rMBP, to achieve different P/L ratios. The samples were incubated for 1 h at room temperature before grid preparation.

#### Negative stain EM

For negatively stained EM, the sample was applied to glow-discharged, thin carbon film-coated copper EM grids and incubated for 1 min. The grid was then blotted with paper, washed with 4 drops of H_2_O, and negatively stained with 2 drops of 2% uranyl acetate. Grids were imaged on Philips CM200 TEM operated at 200 keV or a Tecnai G2 Spirit 120 kV instrument equipped with a Quamesa CCD camera (Olympus Soft Imaging Solutions).

MBP-His reconstituted into lipid vesicles (brain lipids:POPC:cholesterol) was labelled with 5 nm Ni-NTA-Nanogold particles (Nanoprobe). The sample was immobilised on a glow-discharged, carbon-coated electron microscopy grid prior to labelling. The grid was incubated 30 min upside-down on a droplet of 0.02 × Nanogold label solution on parafilm. The grid was washed twice with a buffer containing 20 mM Tris pH 7.5, 200 mM NaCl, 1% glycerol, and 100 mM imidazole, and negatively stained with 2% uranyl acetate.

#### Nanotube preparation

GalCer-DOGS-NTA-Ni nanotubes were generated as previously described^80^. MBP-His and rMBP (0.4 mg ml^−1^) were incubated for 1 h with 0.2 nmol ml^−1^ nanotubes. Samples were imaged with negative stain or cryoEM.

#### Cryo EM grid preparation, imaging, and processing

Approximately 3 µl of the samples (~0.4 mg ml^−1^) were applied to glow-discharged Quantifoil holey carbon grids (R 1.2/1.3, R 2/2, or R3.5/1, Cu 400 mesh, Quantifoil Micro Tools GmbH, Germany). After 2-s blotting, grids were flash frozen in liquid ethane, using an FEI Vitrobot IV (Vitrobot, Maastricht Instruments) with the environmental chamber set at 90% humidity and a temperature of 20 °C. Samples were imaged with FEI Titan Krios TEM operated at 300 keV, and images were recorded using a Gatan K2-Summit direct electron detector. Images were collected manually in electron-counting mode at a nominal magnification of ×22,500 and a calibrated pixel size of 1.3 Å. Each image was dose-fractionated to 40 frames (8 s in total, 0.2-s frames, dose rate 6–7 e^−^/pixel/s). Movie frames were aligned with MotionCorr^81^ and preprocessed on the fly with 2dx_automator^82^. Particles were boxed using e2boxer.py in EMAN2^83^ and further processed by SPRING^84^. In total, from 20 aligned images, 9000 overlapping, CTF-corrected segments with a size of 190 Å × 190 Å were used to calculate 2D class averages.

### Neutron reflectometry

Supported lipid bilayers were prepared onto flat 80 mm × 50 mm × 15 mm Si-crystal blocks (polished by Sil’tronix Silicon Technologies, Archamps, France to a 5 Å RMS roughness tolerance). Three samples were prepared from chloroform-methanol stocks of 1 mg ml^−1^:DMPC, DMPC:DMPG (1:1), and d_54_-DMPC:d_54_-DMPG (1:1). The two leaflets of the bilayer were deposited sequentially using the Langmuir-Blodgett and Langmuir-Schaefer techniques. Deposition was carried out at a surface pressure held constant at 30 mN m^−1^, following the procedure described previously for mixed, charged lipid systems^85, 86^. The blocks were assembled into low-volume measurement flow cells, to enable the *in situ* exchange of solvent and injection of protein samples^87^, and transferred directly to the neutron reflectometer for measurement.

Each data point was collected at incident angles of 0.8° and 3.2°, on the D17 neutron reflectometer at the ILL (Grenoble, France)^88^. A slit geometry was used such that full use could be made of the coherent summing method for processing the data^89^, maximizing both intensity and resolution during time-resolved measurements. The sample temperature was kept at a constant +30 °C throughout. HBS buffer was used for all measurements, prepared at a final concentration of 95% (v/v) deuterium oxide (D_2_O, Sigma-Aldrich) and in H_2_O. The bilayers were characterised, before and after the introduction of rMBP, at three different solvent contrasts, varying the D_2_O-H_2_O volume fraction introduced into the sample cell: (1) 95% D_2_O, (2) water contrast-matched to Si (SMW; 38-to-62 volume ratio of D_2_O/H_2_O), and (3) 100% H_2_O. During the injection of rMBP, in H_2_O, time-resolved measurements were carried out, cycling between the two angles to give a time resolution of ~15 minutes. rMBP was allowed to interact with the membrane for 3 h, until no further changes were seen in the reflectivity. Any excess rMBP was then washed out from the bulk solution by pumping solvent slowly through the sample cell, until the cell volume had been exchanged 20 times.

Data analysis was carried out using custom procedures with the RasCAL software package (https://sourceforge.net/p/rscl/wiki/Home/). A complete description of the theory and procedures involved in neutron reflectivity analysis for biomembrane systems involving small molecule interactions is discussed elsewhere^85^. Briefly, all equilibrium measurements for one sample, before and after addition of rMBP, were fitted simultaneously with the parameters used to describe the silicon oxide thickness, coverage, and interfacial roughness held constant between all the contrasts. The lipid bilayers were analysed using just five parameters, using scattering length densities and a custom-built model, as described previously in detail^86^. The parameters used were; area per lipid molecule (APM), the number of water molecules associated with the headgroups (H_2_O_head_) and the tails (H_2_O_tail_) of each lipid molecule, and the roughness of the system (both local and global). To account for the interaction of rMBP with the bilayer after injection, four different models were used to try and fit the data: (1) A layer of MBP on top of the membrane but with no penetration in to the bilayer; (2) A layer of MBP on top of the membrane and penetrating into the lipid ‘headgroups’ in the outer leaflet of the bilayer, displacing water molecules; (3) A layer of MBP on top of the membrane and full insertion in to the outer leaflet of the bilayer, potentially displacing both lipid and water molecules from this leaflet; (4) A layer of MBP on top of the membrane and full insertion into both leaflets of the bilayer, potentially displacing water and lipid molecules from both. These displacement/insertion models were set up as quantified and described in earlier work^90^; in all cases, a new ‘local outer leaflet’ roughness was introduced to describe the interface between the lipid heads and the MBP layer. The uncertainty on the value of each parameter used to fit the data was calculated by ‘bootstrapping’, a Monte Carlo error analysis approach built in to RasCAL, taking into account the error and instrumental resolution per data point^91^. This error estimation provides an indication of the uniqueness of the applied model within the error bars of the data.

### Data availability

The datasets generated and analysed during the current study are available from the corresponding author on reasonable request.

## Electronic supplementary material

Supplementary Information
