# Supplementary material for: Membrane Association Landscape of Myelin Basic Protein Portrays Formation of the Myelin Major Dense Line
Source: Sci Rep. 2017 Jul 10;7:4974. doi: 10.1038/s41598-017-05364-3 (PMC5504075; doi:10.1038/s41598-017-05364-3)
Supplement: Supplementary file 1 — Supplementary Information [file 41598_2017_5364_MOESM1_ESM.pdf]

Membrane Association Landscape of Myelin Basic Protein Portrays Formation of the Myelin Major Dense Line

Arne Raasakka[1,2], Salla Ruskamo[2], Julia Kowal[3], Robert Barker[4,5], Anne Baumann[1,6], Anne Martel[5], Jussi Tuusa[2], Matti Myllykoski[2], Jochen Bürck[7], Anne S. Ulrich[7,8], Henning Stahlberg[3] & Petri Kursula[1,2]

[1]Department of Biomedicine, University of Bergen, Bergen, Norway
[2]Faculty of Biochemistry and Molecular Medicine & Biocenter Oulu, University of Oulu, Oulu, Finland
[3]Center for Cellular Imaging and NanoAnalytics (C-CINA), Biozentrum, University of Basel, Basel, Switzerland
[4]School of Physical Sciences, University of Kent, Canterbury, Kent, United Kingdom
[5]Institut Laue-Langevin (ILL), Grenoble, France
[6]Division of Psychiatry, Haukeland University Hospital, Bergen, Norway
[7]Institute of Biological Interfaces (IBG-2), Karlsruhe Institute of Technology, Karlsruhe, Germany
[8]Institute of Organic Chemistry, Karlsruhe Institute of Technology, Karlsruhe, Germany

Corresponding author: petri.kursula@uib.no

Supplementary information

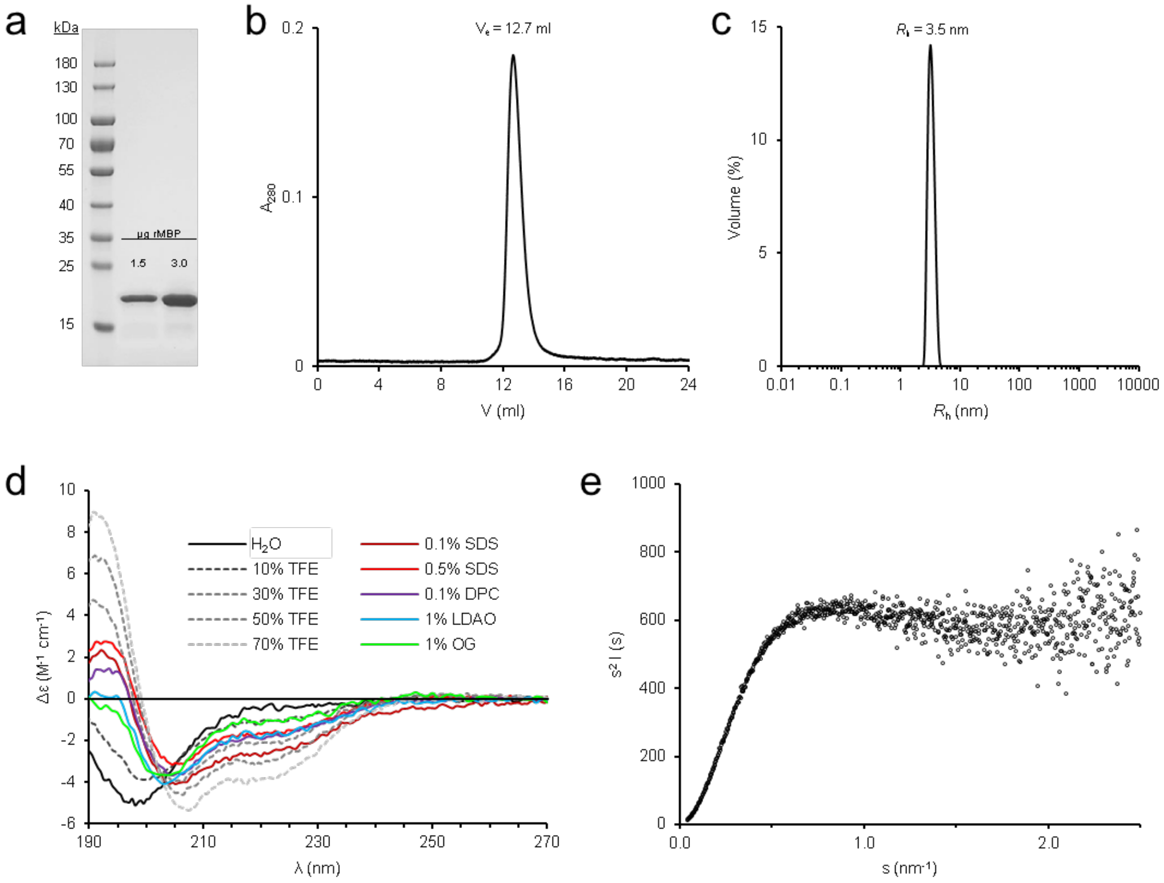

Supplementary Fig. 1. The purity, monodispersity, and folding of rMBP. (a) The purity of rMBP as determined by SDS-PAGE. (b) Size-exclusion chromatogram of rMBP when analysed using a Superdex 75 pg 10/300GL column. (c) Size distribution of rMBP in DLS. (d) The conformational changes of rMBP in TFE and various detergents were followed by CD spectroscopy. (e) rMBP appears mostly elongated based on SAXS data, when plotted in a Kratky plot.

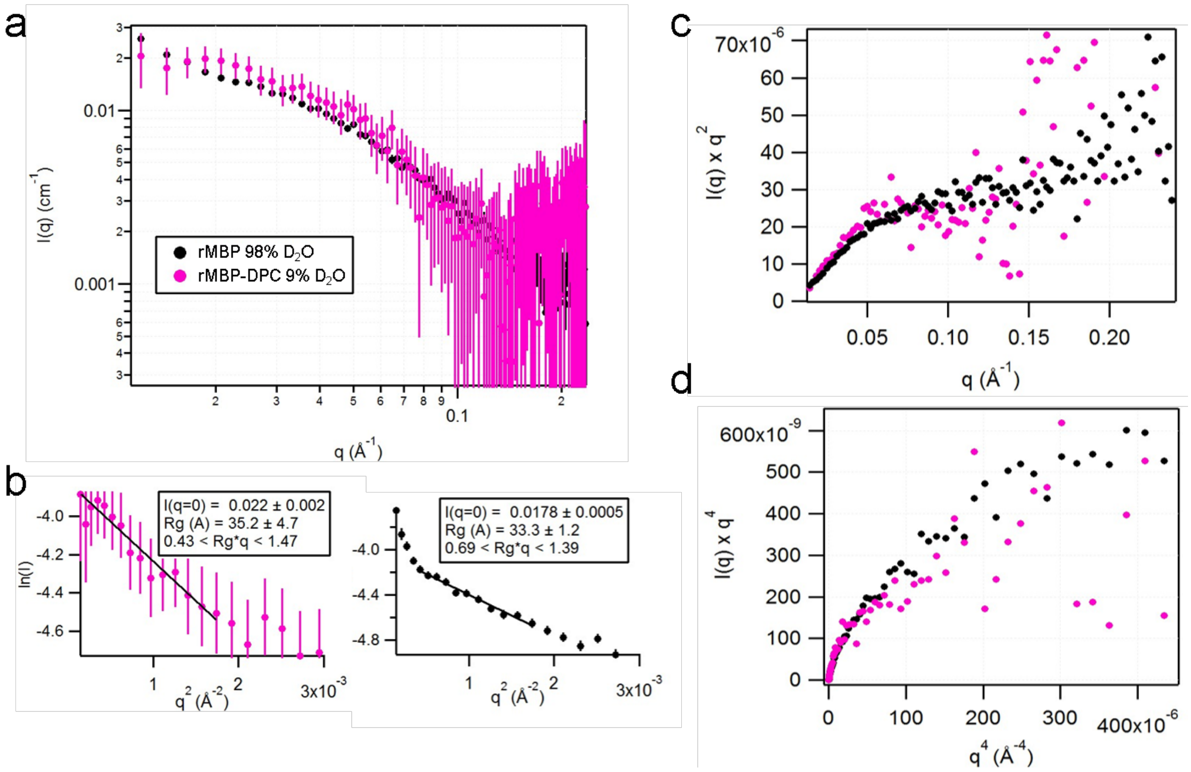

Supplementary Fig. 2. SANS data analysis. (a) Neutron scattering data of rMBP and DPC-bound rMBP. (b) Guinier analysis. (c) Kratky plot. (d) Porod-Debye plot. Comparison of the two datasets suggests that in the presence of DPC, rMBP forms a partially folded structure that represents a micelle-embedded segment, while still remaining mostly extended.

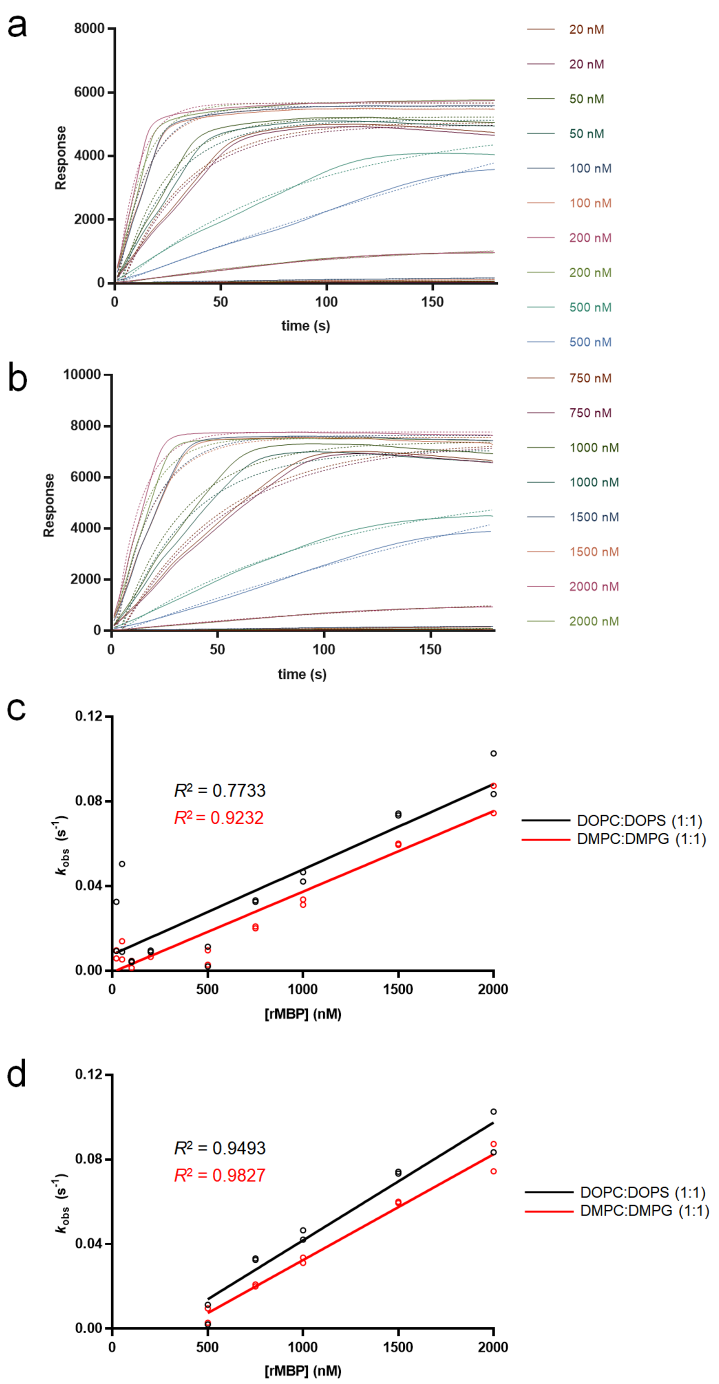

Supplementary Fig. 3. Kinetic analysis of SPR data. The association data (solid lines) of rMBP binding to DOPC:DOPS (a) and DMPC:DMPG (b) vesicles were fitted individually to an exponential one-phase association binding model (dashed lines). The derived k_{obs} values were plotted against rMBP concentration, and the apparent k_{on} and k_{off} values were extracted (see Supplementary Table 2) for DOPC:DOPS and DMPC:DMPG by fitting linear functions to the entire data (c) as well as omitting all datapoints prior to the critical binding concentration of rMBP (d), which results in a better linear function.

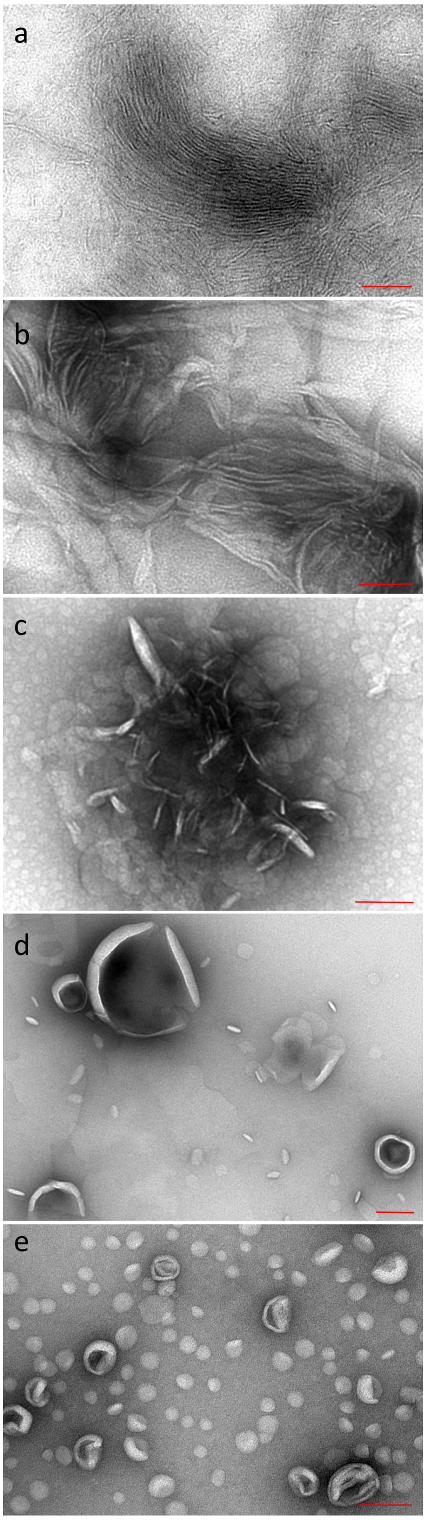

Supplementary Fig. 4. Concentration dependence of membrane stacking induced by rMBP studied by EM. Vesicle aggregation and membrane stacking is very pronounced when rMBP is abundant and gradually decreases together with the concentration of rMBP, as demonstrated by the P/L ratios 1:100 (a), 1:200 (b), 1:500 (c), and 1:1000 (d). Empty vesicles are devoid of aggregation (e). Scale bar: 100 nm.

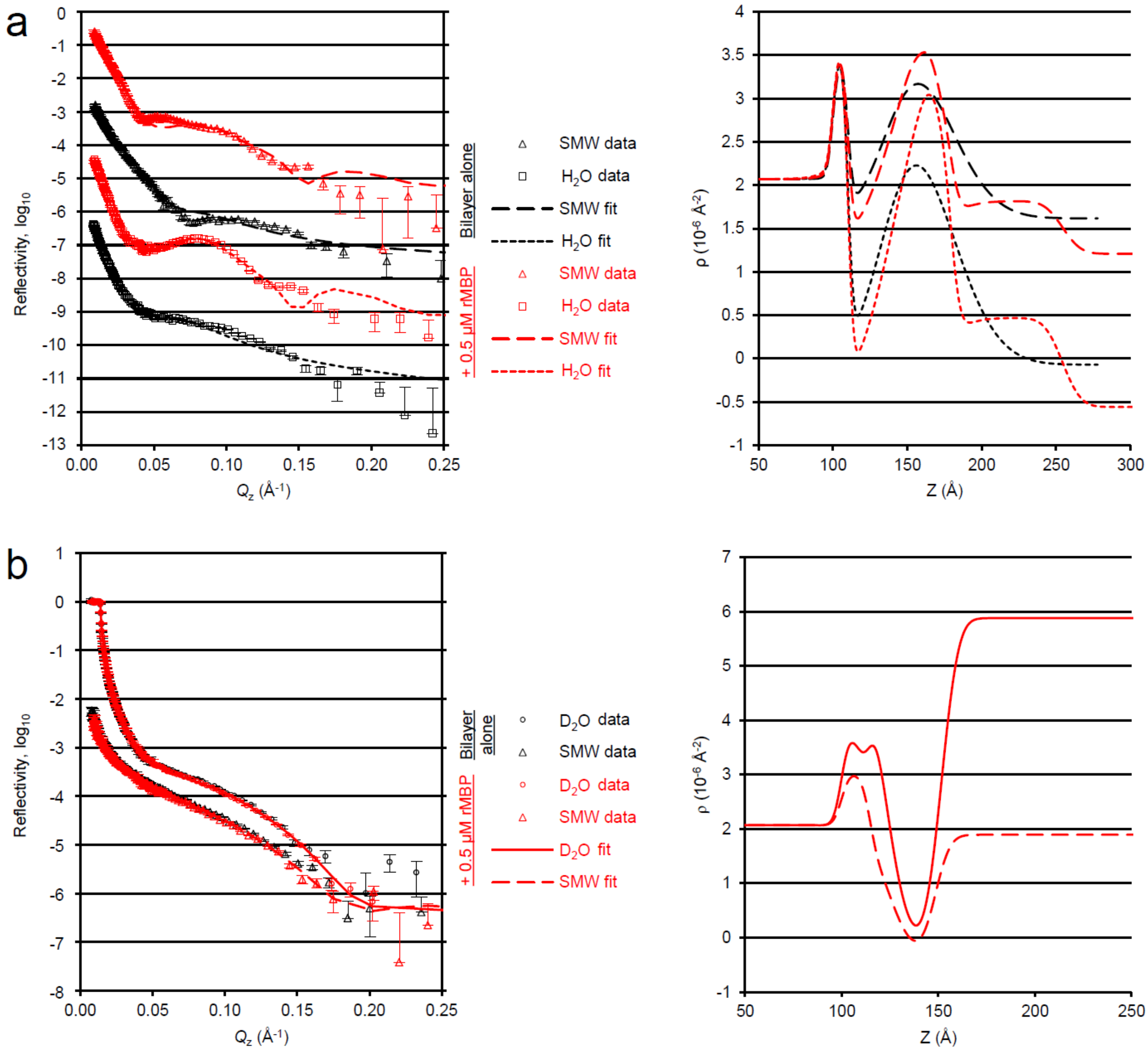

Supplementary Fig. 5. NR control binding experiments. (a) rMBP binds perdeuterated d_{54}-DMPC:d_{54}-DMPG membranes similarly to the hydrogenated membranes. (b) The addition of rMBP to DMPC membranes displays reflectograms that are nearly identical to the naked bilayer, indicating that rMBP is not binding. Data and fits are plotted on the left. All reflectivity curves have been offset for clarity. Scattering length density (ρ) profiles are plotted on the right. The error bars correspond to standard deviation.

Supplementary Table 1. Small-angle scattering data collection and analysis.

**Data collection parameters**			
Method; dataset	SAXS; MBP	SANS; MBP	SANS; MBP + 0.25% DPC
Instrument	P12, PETRAIII, DESY	D22, ILL	D22, ILL
Wavelength (nm)	0.124	0.6	0.6
Angular range (nm^{-1})	0.027 - 4.801	0.12 – 2.364	0.12 – 2.364
Exposure time (s)	0.045	3600	3600
Concentration range (mg ml^{-1})	1.1 - 4.4	2.3	2.3
Temperature (°C)	20	10	10
D_2O contrast (%, v/v)	0	98	9
**Structural parameters**			
I_0 (relative) [from p(r)]	5687		
R_g (nm) [from p(r)]	4.02		
I_0 (relative) [from Guinier]	5582.53	0.0178	0.022
R_g (nm) [from Guinier]	3.68	3.3	3.5
R_g (nm) [from EOM ensemble]	3.96	4.4	3.5
D_{max} (nm) [from GNOM]	17.82	12.0	10.0
D_{max} (nm) [from EOM ensemble]	12.92	13.8	11.2
**Molecular mass determination**			
Molecular mass M_r (kDa) [from I_0 using p(r)]	25.8		
Molecular mass M_r (kDa) [from I_0 using Guinier]	25.4		
Theoretical M_r from sequence (kDa)	18.5	18.5	18.5
**Software**			
Primary data reduction	PRIMUS	GRASP, NCNR SANS	GRASP, NCNR SANS
Data processing	PRIMUS	PRIMUS	PRIMUS
Ab initio analysis	GASBOR		
Conformational ensemble analysis	EOM	EOM	EOM
Validation and averaging	PRIMUS	PRIMUS	PRIMUS
Three-dimensional graphics representation	PyMOL		
**EOM model parameters**			
Conformer #1			
R_g (nm)	5.72	3.38	3.14
D_{max} (nm)	17.81	10.87	9.91
Mass fraction	0.07	0.273	0.647
Conformer #2			
R_g (nm)	4.51	4.75	5.09
D_{max} (nm)	13.31	13.25	14.37
Mass fraction	0.14	0.091	0.182
Conformer #3			
R_g (nm)	4.45	6.42	3.39
D_{max} (nm)	14.20	19.47	11.43
Mass fraction	0.07	0.364	0.182
Conformer #4			
R_g (nm)	3.14	3.57	
D_{max} (nm)	9.34	12.30	
Mass fraction	0.07	0.091	
Conformer #5			
R_g (nm)	3.14	2.92	
D_{max} (nm)	9.47	9.47	
Mass fraction	0.36	0.182	
Conformer #6			
R_g (nm)	3.51		
D_{max} (nm)	11.82		
Mass fraction	0.14		
Conformer #7			
R_g (nm)	4.26		
D_{max} (nm)	12.83		
Mass fraction	0.07		
Conformer #8			
R_g (nm)	6.41		
D_{max} (nm)	18.65		
Mass fraction	0.07		
Total mass fraction of main conformers	0.99	1.00	1.00

Supplementary Table 2. Kinetic parameters derived from rMBP association phase with vesicles.

Vesicle composition	Fitting set 1 [a]			Fitting set 2 [a]		
	k_{on} (nM^{-1} s^{-1}) (× 10^5) [b]	k_{off} (s^{-1}) (× 10^2) [c]	R^2	k_{on} (nM^{-1} s^{-1}) (× 10^5) [b]	k_{off} (s^{-1}) (× 10^2) [c]	R^2
DOPC:DOPS (1:1)	4.0 ± 0.54	0.76 ± 0.52	0.77	5.6 ± 0.45	-1.4 ± 0.58	0.95
DMPC:DMPG (1:1)	3.8 ± 0.27	-0.066 ± 0.26	0.92	5.0 ± 0.23	-1.8 ± 0.30	0.98

[a] Fitting set 1 contains all data points from the linear fit, whereas data points below 500 nM were omitted from Fitting set 2.

[b] Slope of the linear fit function to $k_{obs(on)}$ *vs*. [rMBP].

[c] Y-axis intercept of the linear fit function to $k_{obs(on)}$ *vs*. [rMBP]

Supplementary Table 3. Time-resolved neutron reflectometry parameters.

Parameters		d_{54}-DMPC:d_{54}-DMPG (1:1)		
		Phase 1	Phase 2	Phase 3
Substrate	Oxide thickness (Å)	10 ± 1		
	Oxide coverage (%)	90 ± 4		
	Oxide roughness (Å)	3 ± 1		
	Hydration layer between oxide and bilayer (Å)	25 ± 6		
Bilayer	Bilayer area per molecule ($Å^2$/molecule)	59 ± 7	58 ± 6	53 ± 2
	Water per lipid head	10 ± 2	10 ± 5	10 ± 2
	Water per lipid tail	6 ± 3	7 ± 1	8 ± 2
	Global bilayer roughness (Å)	20 ± 5	24 ± 2	24 ± 2
	Local bilayer inner roughness (Å)	5 ± 3	8 ± 1	12 ± 1
	Local bilayer outer roughness (Å)	1 ± 2	6 ± 2	1 ± 1
rMBP	rMBP in outer heads (%)	0	28 ± 10	48 ±12
	rMBP in outer tails (%)	0	0	20 ± 5
	rMBP layer thickness (Å)	70 ± 20	56 ± 3	52 ± 3
	rMBP layer coverage (%)	25 ± 7	47 ± 10	37 ± 6
	rMBP layer roughness (Å)	58 ± 20	8 ± 3	7 ± 5
